# C_3_H_8_O_2_ Isomers: Insights into Potential Interstellar Species

**DOI:** 10.1021/acs.jpca.4c04804

**Published:** 2024-11-11

**Authors:** Lisset Noriega, Luis Armando González-Ortiz, Filiberto Ortíz-Chi, Alan Quintal, Sandra I. Ramírez, Gabriel Merino

**Affiliations:** † Departamento de Física Aplicada, Centro de Investigación y de Estudios Avanzados, Unidad Mérida, km 6 Antigua Carretera a Progreso, Apdo. Postal 73, Cordemex, 97310 Mérida, Yucatán, México; ‡ Conahcyt-Departamento de Física Aplicada, Cinvestav-IPN, Antigua Carretera a Progreso km 6, Mérida, Yucatán 97310, México; § Centro de Investigaciones Químicas, Universidad Autónoma del Estado de Morelos, Av. Universidad 1001 Chamilpa, Cuernavaca, Morelos C. P. 62209, México

## Abstract

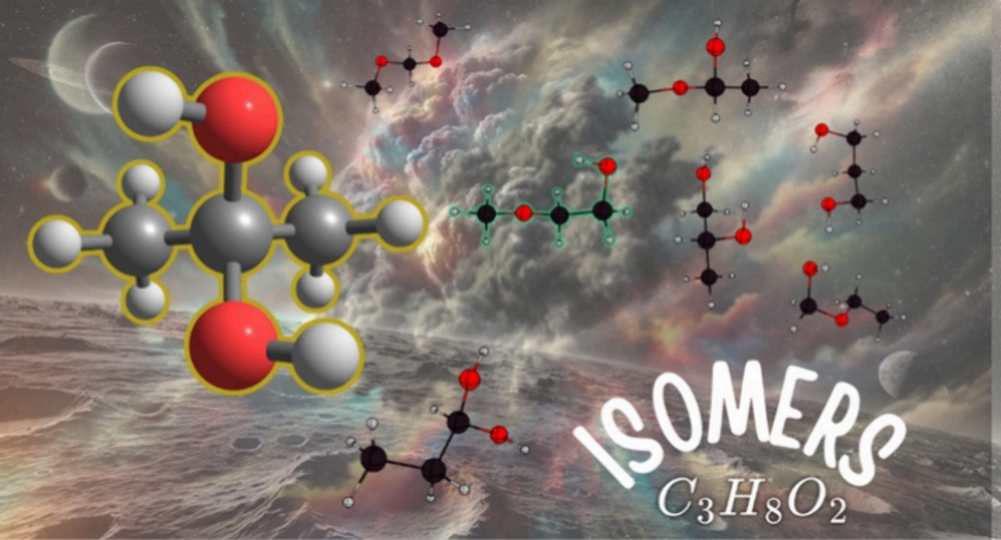

2-Methoxyethanol, with a formula C_3_H_8_O_2,_ was recently identified in the massive protocluster NGC 6334I. However, its structural isomers, 1,2-propanediol and 1,3-propanediol, remain undetected despite extensive searches in the Sgr B2 region. In this study, we explored the potential energy surface of the C_3_H_8_O_2_ system using CCSD(T)/aug-cc-pVTZ//MP2/aug-cc-pVTZ calculations, identifying 11 species, with the geminal diols 2,2-propanediol and 1,1-propanediol as the most stable forms. We examined the gas-phase decomposition barrier of these geminal diols and found that 1,1-propanediol is thermodynamically stable at low temperatures (10–150 K). C_3_H_8_O_2_ isomers with energies below 30 kcal/mol are relevant to the ISM, as they have been identified or tentatively detected in irradiation experiments of ice analogs of CO, H_2_O, and CH_3_OH.

## Introduction

Lattelais et al.^1^ proposed that the most stable molecule is typically the most abundant and, therefore, the most likely to be detected in the interstellar medium (ISM). This principle is supported by examples of complex organic molecules (COMs) in the ISM, such as methyl acetylene in the C_3_H_4_ stoichiometry^2^ and urea in the CH_4_N_2_O one.^3^ However, higher-energy isomers have also been detected in the ISM. For example, propanal, which is 5.4 kcal/mol higher in energy than acetone, and propylene oxide, 30.9 kcal/mol higher,^4^ have both been observed. Similarly, the most stable C_2_H_5_NO isomer,^5^ acetamide, was observed in Sgr B2 in 2006, but the higher-energy isomer *N*-methylformamide was detected in 2019.^6^ San Andrés et al.^7^ also detected N-cyanomethanimine, a higher-energy isomer of C_2_H_2_N_2_, with comparable abundances to its more stable counterpart. These cases challenge the minimum energy principle (MEP), suggesting that other factors beyond energetic must be considered. Based on this, Ellinger et al.^8^ proposed key factors influencing detection of molecules in the ISM: (i) rigid structures tend to have simpler rotational spectra, facilitating detection; (ii) a dipole moment around 2 D enhances detectability; (iii) the molecule should either be the most stable isomer or within 30 kcal/mol of the global minimum; and (iv) molecules weakly adsorbed onto icy surfaces are more likely to exist in the gas phase, aiding detection.

Recently, Hrodmarsson et al.^9^ proposed vacuum ultraviolet photostability as a key factor in molecular detectability, suggesting the nondetection of 2-aminopropionitrile, the most stable C_3_H_6_N_2_ isomer,^10^ could be due to dissociative photoionization. Chemical kinetics also plays a role in the observed abundance ratios, as molecules formed through energetically favorable pathways, especially those with low-energy intermediates, are more likely to be abundant.^7^ Thus, while the global minimum on the Potential Energy Surface (PES) is often the most favorable candidate for detection,^1^ factors such as dipole moment and kinetic stability also influence detectability in the ISM.^8^

Our interest in COMs detectable in the ISM focused on diols, compounds with two hydroxyl groups. Diols have been identified in the ISM and in meteorites such as Murray and Murchison,^11^ hinting at their role in primitive lipid formation on early Earth.^12,13^ For example, 1,2-ethanediol (ethylene glycol) was detected in the ISM in 2002,^14^ despite not being the global minimum on the PES. Another C_2_H_6_O_2_ isomer, methoxymethanol, was found in the ISM in 2017, but it is also not the global minimum,^15^ which is the geminal diol 1,1-ethanediol.^16^ Thus, exploring the PES is critical for assessing the potential presence of a molecule in space.^17,18^

Let us now extend to C_3_H_8_O_2_, which offers more structural possibilities. Will the global minimum still be a geminal diol? Which of these systems can be detected in the ISM? So far, only 2-methoxyethanol has been identified in the massive protocluster NGC 6334I,^19^ making it the most complex methoxy molecule detected in space. Another isomer, 1,2-propanediol, has been found in meteoritic samples,^11^ and laboratory simulations mimicking space conditions–exposing water, methanol, or carbon dioxide ices to ionizing radiation–have produced 1,2-propanediol and 1,3-propanediol.^11,20,21^ However, despite these findings, these diols remain undetected in the ISM. High-level ab initio computations and spectroscopic techniques have revealed the complex conformational landscape of these diols,^22−28^ keeping the search for interstellar C_3_H_8_O_2_ isomers ongoing.

In this work, we report the first systematic exploration of the PES of C_3_H_8_O_2_. Our CCSD(T)/aug-cc-pVTZ//MP2/aug-cc-pVTZ computations reveal a surprising twist: 2,2-propanediol, a geminal diol, is the most stable isomer, 26 kcal/mol lower in energy than 2-methoxyethanol. Even 1,2-propanediol and 1,3-propanediol are less stable by 12.5 and 16.6 kcal/mol, respectively. Since each structural isomer has multiple conformers, we performed an exhaustive search to identify the most stable one and calculated their dipole moment and rotational constants to facilitate future identification.

## Methodology

While manually sketching and analyzing structural combinations is feasible for small molecular systems, it becomes impractical as system complexity increases. SMILES notation provides a systematic way to represent chemical structures, enabling rapid and exhaustive structure generation. Although C_3_H_8_O_2_ is relatively simple, it was chosen to validate the scalability of this method for future studies on more complex molecules. To obtain the isomers of C_3_H_8_O_2_, a system with an Index of Hydrogen Deficiency (IHD) of zero, we first wrote the SMILES strings for the isomers of C_5_H_12_: CCCCC, CCC(C)C, and CC(C)(C)C. We then modified these by replacing two carbon atoms with two oxygen atoms. After eliminating redundant structures, we identified the following isomers: CCCOO, CCOCO, CCOOC, COCCO, COCOC, OCCCO, CCC(O)O, COC(C)O, OCC(C)O, OOC(C)C, and CC(C)(O)O.

We identified 13 C_3_H_8_O_2_ isomers. Since enantiomers share identical properties (e.g., energy, dipole moment, and rotational constants), we selected one representative from each pair, resulting in 11 isomers for further analysis. Initial geometry optimizations were performed using the M06-2X^29^-D3^30^ method with the aug-cc-pVTZ^31^ basis set. These geometries were refined at the MP2^32^ level of theory with the same basis set, and the final energy calculations were conducted using the CCSD(T)/aug-cc-pVTZ//MP2/aug-cc-pVTZ approach. Frequency calculations confirmed that the structures are true minima (all positive frequencies) and identified transition states (TS) characterized by one imaginary frequency. Additionally, Intrinsic Reaction Coordinate (IRC) analysis ensured that each TS connects the appropriate reactants and products. All computations were performed with Gaussian 16.^33^

Next, we explored the possible conformers for each isomer. Using the formula 3^*n*^ (where *n* is the number of dihedral angles, excluding methyl groups), we systematically explored the conformational space with the Global Optimization of Molecular Systems (GLOMOS) software.^34^ This process generates a grid of torsion angles from 0 to 360° in 120° increments. GLOMOS identifies torsion axes (B–C) as bridges in the molecular graph, with A, B, C, and D as consecutive atoms defining the dihedral angle. Each configuration was optimized without constraints. Initially, 247 conformers were identified, which were reduced to 107 after geometry optimization, as some structures collapsed into more stable minima while others were mirror images of existing conformers (Table S1).

## Results and Discussion

**Figure 1:**
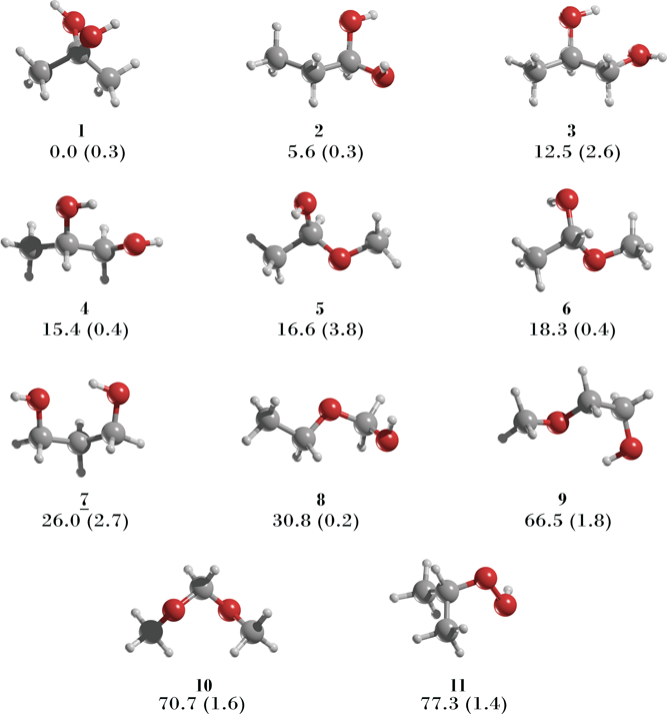
MP2/aug-cc-pVTZ geometries of the most stable conformers of the structural isomers of C_3_H_8_O_2_. Relative energies are in kcal/mol at the CCSD(T)/aug-cc-pVTZ//MP2/aug-cc-pVTZ level. Dipole moments in parentheses are in Debye units. The isomer **7** (underlined) is the one detected in the ISM.

Let us evaluate the likelihood of detecting various C_3_H_8_O_2_ isomers in the ISM. Table 1 and Figure 1 present the most stable conformations identified for all 11 C_3_H_8_O_2_ structural isomers, excluding the stereoisomers of 1,2-propanediol (**3**) and 1-methoxyethanol (**4**). These isomers include four diols, three hydroxy ethers, one diether, and three peroxides. Interestingly, the most stable isomers (**1**, **2**, **3**, and **5**) are diols, exhibiting relative energy differences within 16.6 kcal/mol, with 2,2-propanediol (**1**) being the most stable. In contrast, 1-methoxyethanol (**4**), classified as a hemiacetal, is 15.4 kcal/mol less stable than **1**. The remaining hydroxy ethers (**6** and **7**) are even less stable, with relative energies of 18.3 and 26.0 kcal/mol, respectively. Diether **8** is 30.8 kcal/mol less stable than **1**, while the peroxides (**9**, **10**, and **11**) are the least stable, with an energy difference of about 70 kcal/mol.

**Table 1:** Relative Energies (Δ*E*, kcal/mol) and Dipole Moment (μ, Debye) of the Most Stable Conformer of Each Structural Isomer of C_3_H_8_O_2_^a^

isomer	Δ*E*^b^	μ^c^	formula	name	detected in ice analogs?	detected in meteorites and/or comets?	detected in the ISM?
1	0.0	0.3	CH_3_C(OH)_2_CH_3_	2,2-propanediol	tentative^13^		
2	5.6	0.3	CH_3_CH_2_CH(OH)_2_	1,1-propanediol	tentative^13^		
3	12.5	2.6	CH_3_CHOHCH_2_OH	(2*S*/2*R*))-1,2-propanediol	yes^11,20^	yes^11,35^^d^^,^^e^	
4	15.4	0.4	CH_3_OCH(OH)CH_3_	(1*S*/1*R*)-1-methoxyethanol	yes^36,37^		
5	16.6	3.8	HOCH_2_CH_2_CH_2_OH	1,3-propanediol	yes^11,20,38^		
6	18.3	0.4	CH_3_CH_2_OCH_2_OH	ethoxymethanol	tentative^36^		
7	26.0	2.7	CH_3_OCH_2_CH_2_OH	2-methoxyethanol	tentative^36^	yes^35,39,40^^d^	yes^19^
8	30.8	0.2	CH_3_OCH_2_OCH_3_	dimethoxymethane	yes^41^		
9	66.5	1.8	CH_3_CH(OOH)CH_3_	isopropyl hydroperoxide			
10	70.7	1.6	CH_3_CH_2_CH_2_OOH	propyl hydroperoxide			
11	77.3	1.4	CH_3_CH_2_OOCH_3_	ethylmethyl peroxide			

a Table adapted from references (1,13).

b CCSDT/aug-cc-pVTZ//MP2/aug-cc-pVTZ.

c MP2/aug-cc-pVTZ.

d Detected in Murchison and GRA 06100.

e Detected in the comet 67P/Churymov-Gerasimenko.

### 2,2-Propanediol (**1**)

Isomer **1** has three different conformations within a narrow energy range of 2.7 kcal/mol (Figure S1). The most stable conformation (**1**–**1**) has *C*_2_ symmetry, with antiparallel OH groups and a dipole moment of 0.3 D. In contrast, **1**–**2**, which is 2.5 kcal/mol higher in energy, adopts *C*_s_ symmetry, with both OH groups oriented in the same direction, resulting in a dipole moment of 2.6 D. Finally, in **1**–**3** one OH points toward a methyl group while the other lies between two methyl groups, yielding in a dipole moment of 2.4 D. The distinct dipole moments in these conformers arise from the reorientation of the oxygen lone pairs. We further investigated the population distribution of these conformers at temperatures relevant to nebulae and star-forming regions (10–300 K).^42^ Using the Boltzmann distribution equation (details in Table S2), we determined that only **1**–**1** has a relevant population (98%) within the examined temperature range (10–298 K).

### 1,1-Propanediol (**2**)

Isomer **2** exhibits a more extensive conformational landscape than **1** with 13 different conformations within an energy range of 3.5 kcal/mol. The two low-lying conformers, **2**–**1** and **2**–**2**, have nearly identical energies, differing by only 0.1 kcal/mol, while **2**–**3** is also close in energy, sitting just 0.5 kcal/mol higher than **2**–**1**. At 150 K, the relative populations of these three conformers are 52.5, 37.6, and 9.8%, respectively. Despite this distribution, all three share similar dipole moments around 0.3 D. The remaining conformations (**2**–**4** to **2**–**13**) span an energy range of 2.2–3.5 kcal/mol. Although these conformers possess dipole moments exceeding 2.0 D, which could facilitate detection in the ISM, their relative populations are negligible at temperatures below 150 K.

### 1,2-Propanediol (**3**)

Isomer **3** is of particular interest for astrochemical exploration due to its structural similarity to ethylene glycol and its chirality, a rare property in interstellar molecules. To date, only propylene oxide has been detected as a chiral structure in the ISM,^43^ enhancing **3** as a potential candidate for ISM detection. It has been identified in ice analogs mimicking H_2_O:CH_3_OH mixtures,^20^ in meteorites such as Murchison and Graves Nunataks 06100,^11^ and in comet 67P/Churymov-Gerasimenko, where it was reported to be more abundant than *n*-propanol and 27% more abundant than methanol.^35^

Despite efforts to detect the two most stable conformers of **3** within the Sgr B2(N) molecular cloud as part of the Green Bank Telescope Prebiotic Interstellar Molecule Survey Legacy Project,^28,44^ no spectral lines were identified.^25^ So, protostellar hot cores, such as IRAS 16293–2422,^23,45^ emerge as promising targets for future searches. The detection of molecules like glycolaldehyde, methyl formate, and ethylene glycol in this region^46^ suggests a rich chemical composition that could harbor isomer **3**.

Our computations at the MP2/aug-cc-pVTZ level revealed 22 distinct conformers for **3**, with the eight most stable conformers residing within a narrow energy range of 1.2 kcal/mol. All share a common OCCO skeleton in a gauche configuration, differing primarily in the orientation of their −OH groups. This variation does not significantly impact their dipole moments, as all eight possess values exceeding 2.0 D, enhancing their detectability in the ISM. The least stable conformer, **3**–**22**, is 4.2 kcal/mol less stable than **3**–**1**. At temperatures as low as 10 K, the population distribution heavily favors conformers **3**–**1** and **3**–**2**, accounting for 99.3 and 0.6% of the total population, respectively.

### 1-Methoxyethanol (**4**)

Isomer **4** is notable among the C_3_H_8_O_2_ isomers for being a chiral molecule and a hemiacetal. Hemiacetals are of particular interest in astrochemistry due to their potential role in interstellar sugar synthesis.^47,48^ It can adopt six distinct conformations within an energy range of 4.1 kcal/mol. The most stable conformer, **4**–**1**, has a dipole moment of 0.4 D, comparable to values observed in methoxymethanol, a hemiacetal detected in the ISM.^15^ Conformer **4**–**1** dominates the population at temperatures below 150 K, accounting for roughly 99.0%. This high abundance, along with its dipole moment, makes **4** a promising candidate for future detection.

### 1,3-Propanediol (**5**)

Our PES exploration identified another diol, **5**, which exhibits 22 different conformations. Consistent with previous studies,^49,50^ the two most stable conformers, **5**–**1** and **5**–**2**, are stabilized by intramolecular hydrogen bonding interactions that form a quasi–six-membered ring, contributing to their stability. They are separated by only 0.2 kcal/mol and have dipole moments of 3.6 and 2.9 D, respectively, making both promising candidates for future detection in various regions of the ISM.^51^ Similar to **3**, isomer **5** has been detected in ice analogs mimicking H_2_O:CH_3_OH mixtures.^11,20,38^ However, searches for **5**–**1** and **5**–**2** within the Sgr B2(N) molecular cloud were unsuccessful.^28^

### Ethoxymethanol (**6**)

Isomer **6**, classified as a hydroxyether, can adopt ten different conformations within an energy range of 3.8 kcal/mol. The most populated conformers are **6**–**1** (95.2%) and **6**–**2** (3.3%), with dipole moments of 0.4 and 0.5 D, respectively. Previous studies have tentatively identified **6** by infrared spectroscopy in methanol ices exposed to irradiation, simulating the effects of galactic cosmic rays.^36^ Despite its low dipole moment, which poses challenges for astronomical observations, **6** remains a relevant candidate for future ISM exploration efforts.

### 2-Methoxyethanol (**7**)

Among the C_3_H_8_O_2_ isomers, **7** is the only molecule detected in the ISM. It is energetically less favorable than **1** per 26 kcal/mol and can adopt 12 different conformations within an energy range of 4.1 kcal/mol. Previous studies focused on the four lowest-energy conformers,^52,53^ identifying **7**–**1** as the most stable. This conformer shows a hydrogen bond (2.327 Å) between the hydrogen of the hydroxyl group and the oxygen of the ether moiety. **7**–**1** has a dipole moment of 2.7 D and becomes the only populated conformer at low temperatures (95%). Before its confirmed detection in the ISM in 2024,^19^ isomer **7** was tentatively identified through infrared spectroscopy in methanol ices.^36^ Its presence was also established in comet 67P/Churymov-Gerasimenko (67P), where it exhibits a relative methanol abundance of 27%.

### Diether Dimethoxymethane (**8**)

Isomer **8**, the last identified isomer within an energy range of 30 kcal/mol, can adopt four conformations. The low-lying conformer **8**–**1** exhibits a short C–H···O distance of 1.992 Å, suggesting stabilization from both the anomeric effect and a weak intramolecular C–H···O interaction.^54,55^ Conformer **8**–**1** is the most populated form in the gas phase at 298.15 K, with and abundance of 98.6%, and remains the only one present at temperatures as low as 150 K. The relative abundance of **8** was determined through infrared spectroscopy in experiments simulating interstellar environments, where methanol ice analogs were exposed to UV irradiation, mimicking cosmic radiation effects that can trigger the formation of COMs like **8**. Its proposed formation mechanism involves the recombination of more than two radicals, potentially explaining its detection only after 30 min of irradiation.^41,56^ So, despite this slower formation pathway, **8** emerges as a candidate worthy of further exploration in the ISM due to its presence in simulated interstellar environments.

### Peroxides (**9**–**11**)

Our PES exploration identified isopropyl hydroperoxide (**9**), propyl hydroperoxide (**10**), and ethyl methyl peroxide (**11**) as the least energetically favored molecules, residing 66.5, 70.7, and 77.3 kcal/mol above the global minimum, respectively. Despite their low stability, organic peroxides are key intermediates in gas-phase hydrocarbon oxidation and play a critical role in free radical chain termination and aerosol formation within Earth’s atmosphere.^57^ Isomer **9** has three distinct conformers within an energy range of 0.8 kcal/mol, with **9**–**1** (1.6 D) and **9**–**2** (1.7 D) being the most populated isomers at low temperatures, with relative abundances of 56.7 and 40.5% at 150 K. Isomer **10** has nine different conformations, with the most stable conformers, **10**–**1** (1.4 D) and **10**–**2** (1.8 D), dominating the population at low temperatures (30.8 and 15.7% at 150 K). These two conformers differ by only 0.2 kcal/mol. Isomer **11** possesses three conformers with similar energies (within 0.1 kcal/mol), exhibiting dipole moments of 1.6 (**11**–**1**), 1.3 (**11**–**2**), and 1.5 (**11**–**3**) D, respectively.

Note that these peroxides exhibit dipole moments ranging from 1.3 to 1.9 D, within the sensitivity limits of rotational spectroscopy, making these molecules potential candidates for detection in the ISM. However, organic peroxides have not been identified in the ISM, comets, or meteorites,^35^ leaving their astrochemical significance elusive, partly due to their high reactivity.

### Geminal Diols in Space?

Although geminal diols have not yet been detected in the ISM, they are fascinating targets for astrochemical research. A known decomposition pathway for these molecules involves spontaneous dehydration, yielding aldehydes and water.^58,59^ Laboratory simulations of cold interstellar environments have successfully synthesized and characterized methanediol, the simplest geminal diol, using infrared and mass spectrometry techniques by Zhu et al.^60^ Quantum chemical computations by Kent et al. indicate that methanediol remains stable at temperatures below 100 K under simulated ISM conditions.^61^ Further studies by Kumar and Francisco examined the uncatalyzed dehydration mechanism for methanediol and 1,1-ethanediol,^62^ estimating the dehydration barriers at 42.4 and 40.5 kcal/mol, respectively.

**Figure 2:**
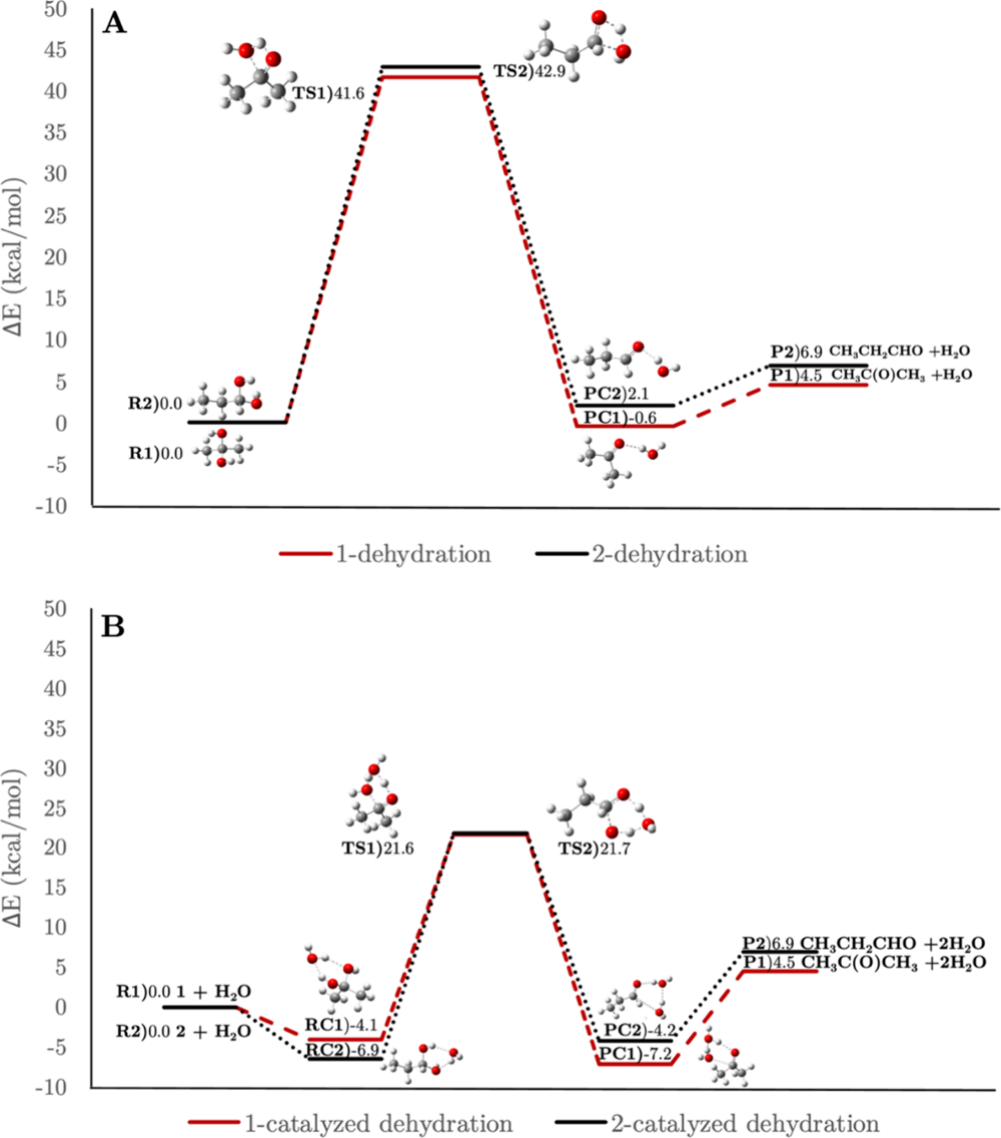
Uncatalyzed dehydration (**A**) and water-catalyzed dehydration (**B**) of isomers **1** and **2** calculated at the CCSDT/aug-cc-pVTZ//MP2/aug-cc-pVTZ (298.15 K, 1 atm) level of theory.

Isomers **1** and **2** were tentatively identified through reflection absorption infrared spectroscopy following the hydroxylation of *n*-propenol and isopropanol, mimicking conditions in dense molecular clouds with temperatures as low as 10 K.^13^ This suggests that these isomers could form in icy environments during the early stages of dark cloud development, but may undergo spontaneous decomposition upon desorption into the gas phase.^63^ To gain further insights into the potential presence of isomers **1** and **2** in the ISM, we investigated the gas-phase uncatalyzed and water-catalyzed dehydration barriers for their two most stable conformers (Figure 2A and Figure 2B, respectively).

In both cases, dehydration involves a transition state where a hydrogen atom is transferred between the hydroxyl groups. Diol **1**, with the hydroxyl groups on a secondary carbon atom, undergoes uncatalyzed dehydration to form acetone and water, with an energy barrier of 41.6 kcal/mol. The dehydration reaction of **1** is exergonic within the temperature range of 150–298 K (Table 2). The presence of a water molecule significantly reduces the barrier to 25.7 kcal/mol, suggesting that **1** is highly susceptible to decomposition into acetone and water under ISM conditions. Water in the ISM could facilitate this dissociation, complicating detection since rotational spectroscopy is not well-suited for identifying such species, particularly in icy environments. Isomer **2** exhibits a slightly higher dehydration barrier of 42.9 kcal/mol for uncatalyzed dehydration compared to **1**, resulting in the formation of propanal and water complex (PC2). Unlike diol **1**, the uncatalyzed dehydration of diol **2** is endergonic within the temperature range of 10–140 K. The presence of a water molecule as a catalyst significantly lowers the dehydration barrier to 28.3 kcal/mol. These results suggest that diol **2** is more likely to survive in the ISM than **1**, potentially increasing the chances of detecting **2** with rotational spectroscopy.

**Table 2:** Free Energies for Uncatalyzed and Water-Catalyzed Dehydration (kcal/mol) of Isomers 1 and 2, at Different Temperatures Calculated at MP2/aug-cc-pVTZ

	10 K	50 K	100 K	140 K	150 K	298 K
**1** dehydration						
Δ*G*_TS1_	40.3	40.2	40.2	40.1	40.1	39.9
Δ*G*_PC1_	–0.4	–0.6	–1.0	–1.6	–1.7	–4.2
Δ*G*_P1_	4.6	3.5	1.7	0.0	–0.4	–7.0
**1** water-catalyzed dehydration						
Δ*G*_RC1_	–3.9	–3.0	–1.6	–0.5	–0.2	4.2
Δ*G*_TS1_	19.8	20.7	22.1	23.4	23.7	28.9
Δ*G*_PC1_	–7.1	–6.3	–5.3	–4.6	–4.4	–2.2
Δ*G*_P1_	4.6	3.5	1.7	0.0	–0.4	–7.0
**2** dehydration						
Δ*G*_TS2_	41.4	41.4	41.4	41.4	41.4	41.4
Δ*G*_PC2_	2.1	2.0	1.7	1.4	1.3	–0.5
Δ*G*_P2_	6.8	5.8	4.2	2.8	2.4	–3.2
**2** water-catalyzed dehydration						
Δ*G*_RC2_	–6.5	–5.6	–4.1	–2.9	–2.6	1.8
Δ*G*_TS2_	19.7	20.6	22.1	23.5	23.8	29.2
Δ*G*_PC2_	–4.0	–3.2	–2.2	–1.4	–1.2	1.2
Δ*G*_P2_	6.8	5.8	4.2	2.8	2.4	–3.2

The energy barrier for a water-catalyzed reaction decreases by up to 20 kcal/mol. Simulating icy grains by adding water molecules could further lower the barrier, as in the isomerization of HCN to CNH, where the barrier was significantly reduced in ice grain simulations.^64^ Thus, geminal diols in the ISM warrant further investigation through ice surface simulations, which may lower the barrier and facilitate their decomposition. While this is beyond the scope of our current work, it represents an important direction for future research.

### Proposed Reaction Pathways for the Most Stable Isomers

The eight most stable C_3_H_8_O_2_ isomers can form in space through radical–radical recombination. As one of the reviewers noted, isomer **7**, characterized by its relatively high dipole moment, follows a favorable reaction pathway, likely originating from the reaction between the 2-hydroxyethyl radical (·CH_2_CH_2_OH) and the methoxy radical (CH_3_O·), enhancing its detectability in the ISM. The CH_3_O· radical has been detected in the ISM,^65^ while ·CH_2_CH_2_OH could form from ethanol photodissociation or the reaction between hydroxyl radical (·OH) and ethylene (C_2_H_4_).^19^ In contrast, isomer **3** is hypothesized to form from the recombination of the hydroxymethyl radical (·CH_2_OH) and 1-hydroxyethyl radical (CH_3_CH(OH)·), both intermediates in ISM reactions.^66^ Wang et al.^37^ showed the synthesis of racemic isomer **4** in interstellar ice analogs using acetaldehyde and methanol, suggesting that hemiacetal formation from interstellar aldehydes and alcohols is plausible. Isomer **4** may also form through the recombination of methoxy and CH_3_CH(OH)· radicals. Isomer **5** could arise from the recombination of hydroxymethyl and 2-hydroxyethyl radicals, similar to isomer **3**. Lastly, **8** may result from the combination of methoxy and methoxymethyl (·CH_2_OCH_3_) radicals, both proposed as intermediates in star-forming regions.^66^

### Rotational Constants

Rotational spectroscopy has played a critical role in the detection of over 320 ISM species.^67^ For **3**, experimental rotational constants exist for conformers **3**–**1** to **3**–**7**,^22,24−26^ while conformers **5**–**1** and **5**–**2** have reported rotational constants.^27,28^ Isomer **8**–**1**, exhibiting *C*_2_ symmetry, has reported rotational constants obtained using millimeter wave-free jet absorption spectroscopy, indicating a rotational temperature between 10 and 20 K.^68^ However, data for isomers **1**, **2**, **4**, **6**, and **9**–**11** are currently unavailable. We calculated the rotational constants at the MP2/aug-cc-pVTZ level and compared them with these experimental data, finding percentage differences ranging from 0.07 to 2.5% (Table S3).

However, data for isomers **1**, **2**, **4**, **6**, and **9**–**11** are currently unavailable. The rotational constants for isomer **1** were obtained at the CCSD(T)/ cc-PVTZ level, achieving excellent agreement with MP2/aug-cc-pVTZ calculations. The difference in rotational constants is less than 0.4%, providing reliable values for the other calculated isomers. In this regard, these MP2-calculated values are expected to be used as a guide for future experimental investigations. In Table 3, we present the computed rotational constants for the most stable conformer of each isomer. The complete set of rotational constants for all identified conformers from the PES exploration can be found in Table S4.

**Table 3:** Calculated Equilibrium Rotational Constants (A_e_, B_e_, C_e_, MHz), Dipole Moment Components (μ_a_, μ_b_, μ_c_, Debye), and Permanent Dipole Moment (μ, Debye) at the MP2/aug-cc-pVTZ Level of Theory for the Lowest-Energy Conformer of the C_3_H_8_O_2_ Structural Isomers

isomer	A_e_	B_e_	C_e_	μ_a_	μ_b_	μ_c_
**1**–**1**	5102.1	4843.7	4763.5	0.0	–0.26	0.0
**2**–**1**	8634.8	3585.8	2795.5	–0.24	–0.05	–0.13
**3**–**1**	8643.1	3672.6	2818.1	1.27	–2.05	0.51
**4**–**1**	8420.2	3949.2	2974.2	0.05	–0.35	–0.10
**5**–**1**	7712.7	3964.7	2894.3	3.05	1.56	1.14
**6**–**1**	14447.4	2600.2	2436.8	–0.37	0.12	–0.14
**7**–**1**	12973.0	2777.7	2496.1	2.16	1.22	0.13
**8**–**1**	10039.7	3366.0	3144.3	0.0	–0.28	0.0
**9**–**1**	7919.8	3889.4	2869.3	–0.74	–0.35	1.42
**10**–**1**	8902.9	3181.0	2964.4	–0.25	1.29	0.60
**11**–**1**	16712.7	2479.6	2428.6	0.30	0.75	1.43

All identified species possess nonzero dipole moments, making them rotationally active. Additionally, all isomers are asymmetric top molecules (A_e_ > B_e_ > C_e_) and have at least one nonzero dipole moment in the *x*, *y*, or *z* direction.

## Conclusions

This study presents the first comprehensive exploration of the C_3_H_8_O_2_ potential energy surface. Notably, 2-methoxyethanol is currently the only detected member of this family in the interstellar medium. However, the identification of at least six isomers with higher stability than 2-methoxyethanol suggests a high probability of discovering additional isomers in the ISM.

Observations in other CHO systems support this claim. For instance, three out of six possible isomers have been identified in the C_2_H_4_O system, while the C_2_H_4_O_2_ and C_3_H_6_O_2_ systems have yielded detections of four and three isomers, respectively. These patterns highlight the potential for enriching the C_3_H_8_O_2_ catalog within the ISM.

Our investigation identifies promising candidates for further characterization, including 1,1-propanediol, 1,2-propanediol, 1,3-propanediol, and 1-methoxyethanol. The astrochemical significance of C_3_H_8_O_2_ isomers within the 30 kcal/mol energy range is underscored by their tentative or confirmed identification in ice analog irradiation experiments involving CH_3_OH and H_2_O. These species include two geminal diols, two diols, one hemiacetal, two hydroxy-ethers, and one diether.

Could geminal diols be viable in space? Their thermodynamic stability and resistance to uncatalyzed dehydration suggest this is plausible, indicating potential detectability via infrared spectroscopy. However, further studies, particularly ice grain simulations, are needed to fully support this hypothesis. The detection of **3** and **5** in the ISM is promising due to their enhanced stability compared to **7**, strong dipole moments in their most stable conformers, and the availability of their experimental rotational spectra. These factors position **3** and **5** as strong candidates for targeted searches in ISM regions with oxygen-bearing complex organic molecules. So, this study encourages further astronomical observations and chemical analyses to explore the presence and behavior of these C_3_H_8_O_2_ isomers in space.

## Supporting Information Available

Number of conformers identified theoretically by GLOMOS, along with the final count of conformers after optimization for each isomer of C_3_H_8_O_2_; Boltzmann distribution of the conformers of C_3_H_8_O_2_ at different temperatures at CCSD(T)/aug-cc-pVTZ//MP2/aug-cc-pVTZ level; difference (%) between experimental rotational constants and theoretical rotational constants calculated at MP2/aug-cc-pVTZ level of theory; predicted equilibrium rotational constants, dipole moment components, and dipole moment at MP2/aug-cc-pVTZ of all conformers of C_3_H_8_O_2_, as well as their relative energy (Δ*E*, in kcal/mol) at the CCSD(T)/aug-ccpVTZ//MP2/aug-cc-pVTZ level; figures depicting all conformers of each structural isomer of C_3_H_8_O_2_ identified in the PES exploration; and Cartesian coordinates of the transition states and all the conformers identified (PDF)
