# Supplementary material for: C3H8O2 Isomers: Insights into Potential Interstellar Species
Source: J Phys Chem A. 2024 Nov 11;128(46):9964–71. doi: 10.1021/acs.jpca.4c04804 (PMC11586899; doi:10.1021/acs.jpca.4c04804)
Supplement: Supplementary file 1 — jp4c04804_si_001.pdf [file jp4c04804_si_001.pdf]

SUPPLEMENTAL MATERIAL
FOR

$C_3H_8O_2$ Isomers: Insights into Potential Interstellar Species

Lisset Noriega,[1*] Luis Armando González-Ortiz,[1] Filiberto Ortíz-Chi,[2] Alan Quintal,[1] Sandra I. Ramírez,[3] and Gabriel Merino.[1*]

[1]Departamento de Física Aplicada, Centro de Investigación y de Estudios Avanzados, Unidad Mérida, km 6 Antigua Carretera a Progreso, Apdo. Postal 73, Cordemex 97310, Mérida, Yucatán, México.

[2] Conahcyt-Departamento de Física Aplicada, Cinvestav-IPN, Antigua Carretera a Progreso km 6, Mérida, Yucatán, 97310, México.

[3] Centro de Investigaciones Químicas, Universidad Autónoma del Estado de Morelos, Av. Universidad 1001 Chamilpa, Cuernavaca, Morelos, C. P. 62209, México

Email: gmerino@cinvestav.mx

lisset.noriega@cinvestav.mx

Table S1. Number of conformers identified theoretically (#conf), identified by GLOMOS (initial #conf) and final number of conformers after optimization (Final # conf) for each isomer of $C_3H_8O_2$.

Isomer	# conf	Initial# conf	Final # conf
1	9	6	3
2	27	26	13
3	27	27	22
4	9	9	6
5	81	78	22
6	27	27	10
7	27	26	12
8	9	6	4
9	9	8	3
10	27	27	9
11	9	7	3

Table S2. Boltzman distribution of the conformers of $C_3H_8O_2$ at different temperatures. The relative energy considered was at the CCSD(T)/aug-cc-pVTZ//MP2/aug-cc-pVTZ.

Conformer	298 K	150 K	140 K	100 K	50 K	10 K
1-1	0.982	1.000	1.000	1.000	1.000	1.000
1-2	0.007	0.000	0.000	0.000	0.000	0.000
1-3	0.010	0.000	0.000	0.000	0.000	0.000
2-1	0.421	0.525	0.536	0.593	0.729	0.994
2-2	0.356	0.376	0.374	0.359	0.266	0.006
2-3	0.181	0.098	0.089	0.048	0.005	0.000
2-4	0.010	0.000	0.000	0.000	0.000	0.000
2-5	0.009	0.000	0.000	0.000	0.000	0.000
2-6	0.007	0.000	0.000	0.000	0.000	0.000
2-7	0.002	0.000	0.000	0.000	0.000	0.000
2-8	0.002	0.000	0.000	0.000	0.000	0.000
2-9	0.004	0.000	0.000	0.000	0.000	0.000
2-10	0.004	0.000	0.000	0.000	0.000	0.000
2-11	0.002	0.000	0.000	0.000	0.000	0.000
2-12	0.001	0.000	0.000	0.000	0.000	0.000
2-13	0.001	0.000	0.000	0.000	0.000	0.000
3-1	0.241	0.370	0.385	0.465	0.661	0.993
3-2	0.204	0.264	0.269	0.281	0.242	0.006
3-3	0.172	0.189	0.187	0.170	0.088	0.000
3-4	0.104	0.069	0.064	0.038	0.004	0.000
3-5	0.104	0.069	0.064	0.038	0.004	0.000
3-6	0.053	0.018	0.015	0.005	0.000	0.000
3-7	0.045	0.013	0.011	0.003	0.000	0.000
3-8	0.032	0.007	0.005	0.001	0.000	0.000
3-9	0.007	0.000	0.000	0.000	0.000	0.000
3-10	0.006	0.000	0.000	0.000	0.000	0.000
3-11	0.006	0.000	0.000	0.000	0.000	0.000
3-12	0.005	0.000	0.000	0.000	0.000	0.000
3-13	0.005	0.000	0.000	0.000	0.000	0.000
3-14	0.004	0.000	0.000	0.000	0.000	0.000
3-15	0.004	0.000	0.000	0.000	0.000	0.000
3-16	0.004	0.000	0.000	0.000	0.000	0.000
3-17	0.003	0.000	0.000	0.000	0.000	0.000
3-18	0.001	0.000	0.000	0.000	0.000	0.000
3-19	0.001	0.000	0.000	0.000	0.000	0.000
3-20	0.001	0.000	0.000	0.000	0.000	0.000
3-21	0.000	0.000	0.000	0.000	0.000	0.000
3-22	0.000	0.000	0.000	0.000	0.000	0.000
4-1	0.883	0.993	0.995	1.000	1.000	1.000
4-2	0.050	0.003	0.002	0.000	0.000	0.000
4-3	0.050	0.003	0.002	0.000	0.000	0.000
4-4	0.013	0.000	0.000	0.000	0.000	0.000
4-5	0.003	0.000	0.000	0.000	0.000	0.000
4-6	0.001	0.000	0.000	0.000	0.000	0.000
5-1	0.389	0.627	0.646	0.726	0.882	1.000
5-2	0.278	0.321	0.315	0.266	0.118	0.000
5-3	0.072	0.022	0.018	0.005	0.000	0.000
5-4	0.051	0.011	0.009	0.002	0.000	0.000
5-5	0.037	0.006	0.004	0.001	0.000	0.000
5-6	0.037	0.006	0.004	0.001	0.000	0.000
5-7	0.019	0.001	0.001	0.000	0.000	0.000
5-8	0.019	0.001	0.001	0.000	0.000	0.000
5-9	0.019	0.001	0.001	0.000	0.000	0.000
5-10	0.013	0.001	0.000	0.000	0.000	0.000
5-11	0.011	0.001	0.000	0.000	0.000	0.000
5-12	0.011	0.001	0.000	0.000	0.000	0.000
5-13	0.009	0.000	0.000	0.000	0.000	0.000
5-14	0.006	0.000	0.000	0.000	0.000	0.000

5-15	0.005	0.000	0.000	0.000	0.000	0.000
5-16	0.005	0.000	0.000	0.000	0.000	0.000
5-17	0.005	0.000	0.000	0.000	0.000	0.000
5-18	0.005	0.000	0.000	0.000	0.000	0.000
5-19	0.002	0.000	0.000	0.000	0.000	0.000
5-20	0.004	0.000	0.000	0.000	0.000	0.000
5-21	0.003	0.000	0.000	0.000	0.000	0.000
5-22	0.000	0.000	0.000	0.000	0.000	0.000
6-1	0.725	0.952	0.963	0.992	1.000	1.000
6-2	0.134	0.033	0.026	0.006	0.000	0.000
6-3	0.081	0.012	0.009	0.001	0.000	0.000
6-4	0.035	0.002	0.001	0.000	0.000	0.000
6-5	0.015	0.000	0.000	0.000	0.000	0.000
6-6	0.003	0.000	0.000	0.000	0.000	0.000
6-7	0.002	0.000	0.000	0.000	0.000	0.000
6-8	0.002	0.000	0.000	0.000	0.000	0.000
6-9	0.002	0.000	0.000	0.000	0.000	0.000
6-10	0.001	0.000	0.000	0.000	0.000	0.000
7-1	0.863	0.990	0.993	0.999	1.000	1.000
7-2	0.081	0.009	0.006	0.001	0.000	0.000
7-3	0.009	0.000	0.000	0.000	0.000	0.000
7-4	0.015	0.000	0.000	0.000	0.000	0.000
7-5	0.011	0.000	0.000	0.000	0.000	0.000
7-6	0.008	0.000	0.000	0.000	0.000	0.000
7-7	0.005	0.000	0.000	0.000	0.000	0.000
7-8	0.003	0.000	0.000	0.000	0.000	0.000
7-9	0.002	0.000	0.000	0.000	0.000	0.000
7-10	0.002	0.000	0.000	0.000	0.000	0.000
7-11	0.001	0.000	0.000	0.000	0.000	0.000
7-12	0.001	0.000	0.000	0.000	0.000	0.000
8-1	0.986	1.000	1.000	1.000	1.000	1.000
8-2	0.012	0.000	0.000	0.000	0.000	0.000
8-3	0.001	0.000	0.000	0.000	0.000	0.000
8-4	0.000	0.000	0.000	0.000	0.000	0.000
9-1	0.485	0.567	0.576	0.619	0.732	0.994
9-2	0.409	0.405	0.402	0.374	0.268	0.006
9-3	0.106	0.028	0.023	0.007	0.000	0.000
10-1	0.209	0.308	0.323	0.419	0.743	1.000
10-2	0.149	0.157	0.157	0.153	0.099	0.000
10-3	0.126	0.112	0.110	0.093	0.036	0.000
10-4	0.126	0.112	0.110	0.093	0.036	0.000
10-5	0.126	0.112	0.110	0.093	0.036	0.000
10-6	0.126	0.112	0.110	0.093	0.036	0.000
10-7	0.106	0.080	0.077	0.056	0.013	0.000
10-8	0.023	0.004	0.003	0.001	0.000	0.000
10-9	0.010	0.001	0.001	0.000	0.000	0.000
11-1	0.372	0.412	0.417	0.453	0.578	0.987
11-2	0.314	0.294	0.291	0.274	0.211	0.006
11-3	0.314	0.294	0.291	0.274	0.211	0.006

Table S3. Difference (%) between experimental rotational constants and theoretical rotational constants calculated at MP2/aug-cc-pVTZ level of theory.

	Exp.	Theory	Diff %	Ref.
	Isomer 3-1			
A	8572.05737	8643.10	-0.82	Bossa, J. B.; Ordu, M. H.; Müller, H. S. P.; Lewen, F.; Schlemmer, S. *Astron. Astrophys.* **2014**, *570*, A12.
B	3640.09966	3672.63	-0.88	
C	2790.97277	2818.05	-0.96	
	Isomer 3-2			
A	8393.40092	8608.52	-2.50	Bossa, J. B.; Ordu, M. H.; Müller, H. S. P.; Lewen, F.; Schlemmer, S. *Astron. Astrophys.* **2014**, *570*, A12.
B	3648.55961	3630.06	0.51	
C	2778.30213	2802.32	-0.86	
	Isomer 3-3			
A	8536.77186	8451.83	1.01	Arenas, B. E.; Gruet, S.; Steber, A. L.; Schnell, M. *J. Mol. Spectrosc.* **2017**, *337*, 9–16.
B	3604.19208	3678.89	-2.03	
C	2778.33752	2802.58	-0.87	
	Isomer 3-4			
A	*8327.5924*	8371.48	-0.52	Arenas, B. E.; Gruet, S.; Steber, A. L.; Schnell, M. *J. Mol. Spectrosc.* **2017**, *337*, 9–16.
B	*3641.99952*	3674.59	-0.89	
C	*2776.90418*	2800.99	-0.86	
	Isomer 3-5			
A	*6642.44848*	6672.31	-0.45	Arenas, B. E.; Gruet, S.; Steber, A. L.; Schnell, M. *J. Mol. Spectrosc.* **2017**, *337*, 9–16.
B	*4163.594*	4213.21	-1.18	
C	*3365.3638*	3407.20	-1.23	
	Isomer 3-6			
A	6627.612	6659.25	-0.48	Lovas, F. J.; Plusquellic, D. F.; Pate, B. H.; Neill, J. L.; Muckle, M. T.; Remijan, A. J. *J. Mol. Spectrosc.* **2009**, *257*(1), 82–93
B	4146.287	4192.66	-1.11	
C	3363.345	3407.76	-1.30	
	Isomer 3-7			
A	*6634.7623*	6654.06	-0.29	Arenas, B. E.; Gruet, S.; Steber, A. L.; Schnell, M. *J. Mol. Spectrosc.* **2017**, *337*, 9–16.
B	*4160.63332*	4217.74	-1.35	
C	*3377.90772*	3424.70	-1.37	
	Isomer 5-1			
A	7698.6443	7712.67	-0.18	Smirnov, I. A.; Alekseev, E. A.; Piddyachiy, V. I.; Ilyushin, V. V.; Motiyenko, R. A. *J. Mol. Spectrosc.* **2013**, *293–294*, 33–37
B	3893.5572	3964.72	-1.79	
C	2854.8845	2894.25	-1.36	
	Isomer 5-2			
A	7586.5999	7581.42	0.07	Smirnov, I. A.; Alekseev, E. A.; Piddyachiy, V. I.; Ilyushin, V. V.; Motiyenko, R. A. *J. Mol. Spectrosc.* **2013**, *293–294*, 33–37
B	3881.73578	3960.16	-1.98	
C	2847.0134	2888.07	-1.42	
	Isomer 8.1			

A	*10147.702*	10039.67	1.08	Favero, L. B.; Caminati, W.; Velino, B. *Phys. Chem. Chem. Phys.* **2003**, *5*(21), 4776–4779
B	*3306.749*	3365.98	-1.76	
C	*3089.136*	3144.31	-1.75	

Table S4. Predicted equilibrium rotational constants (A_e,B_e,C_e, MHz), dipole moment components (μ_a,μ_b,μ_c, Debye), permanent dipole moment (μ, Debye) and quartic centrifugal distortion constants (Δ_J, Δ_K, Δ_{JK}, δ_J, δ_K, kHz) at MP2/aug-cc-pVTZ of all conformers of $C_3H_8O_2$ and their relative energy (ΔE, kcal/mol) at CCSD(T)/aug-cc-pVTZ//MP2/aug-cc-pVTZ.

#	Add. label	ΔE	A_e	B_e	C_e	μ_a	μ_b	μ_c	μ	Δ_J	Δ_K	Δ_{JK}	δ_J	δ_K
1-1	*trans*	0	5102.05	4843.67	4763.52	0	-0.26	0	0.26	1.393	0.803	-0.709	0.159	1.43
1-2	*cis*	2.5	5087.99	4829.59	4766.83	-2.47	0	-0.65	2.56	1.074	-2.714	2.776	-0.005	2.625
1-3	*trans'*	2.7	5055.53	4890.44	4738.32	-1.52	1.59	0.97	2.40	1.377	0.357	-0.304	0.147	-0.047
2-1	*I*	5.6	8634.82	3585.77	2795.46	-0.24	-0.05	-0.13	0.28	0.761	1.757	4.778	0.181	2.912
2-2	*II*	5.7	8464.30	3615.32	2791.61	-0.21	-0.05	-0.12	0.25	0.748	0.075	5.423	0.173	3.025
2-3	*III*	6.1	6905.05	4061.34	3378.09	0.34	-0.08	-0.02	0.35	1.547	-8.787	10.184	0.177	0.507
2-4	*IV*	7.8	8438.14	3643.19	2794.79	0.56	0.86	2.15	2.38	0.76	-1.912	8.419	0.187	4.219
2-5	*V*	7.9	8514.80	3634.63	2786.66	-0.93	0.98	1.84	2.28	0.802	2.29	5.561	0.199	3.336
2-6	*VI*	8.0	8782.96	3573.27	2783.03	-0.04	-1.17	1.85	2.19	0.745	1.361	5.582	0.173	3.16
2-7	*VII*	8.3	6837.89	4155.00	3392.70	-2.09	0	1.26	2.44	1.614	-10.052	11.328	0.208	-1.551
2-8	*VIII*	8.3	8326.46	3606.19	2786.17	-1.64	-1.20	-1.94	2.81	0.735	-0.149	5.578	0.169	2.657
2-9	*IX*	8.3	6930.13	4073.72	3396.16	0.8	-1.15	1.75	2.24	1.637	-6.76	8.349	0.186	-0.317
2-10	*X*	8.4	8522.97	3600.50	2783.09	-1.46	-1.99	-0.85	2.61	0.762	1.832	4.511	0.178	2.761
2-11	*XI*	8.7	8650.42	3564.65	2776.07	-2.63	-0.04	-0.62	2.70	0.752	1.922	4.644	0.178	2.727
2-12	*XII*	9.1	6799.85	4022.10	3315.72	2.93	0	-0.63	3.00	1.387	-10.113	11.34	0.166	1.492
2-13	*XIII*	9.1	6896.68	4035.68	3377.51	-2.14	1.31	0.61	2.58	1.623	-7.117	8.297	0.177	1.058
3-1	*tG'g*	12.5	8643.10	3672.63	2818.05	1.27	-2.05	0.51	2.46	0.719	2.974	5.559	0.155	3.164
3-2	*g'G'g*	12.6	8608.52	3630.06	2802.32	-0.41	-1.87	1.52	2.44	0.714	2.991	5.665	0.144	3.117
3-3	*gG't*	12.7	8451.83	3678.89	2802.58	-2.65	0.19	-0.6	2.72	0.772	3.439	4.877	0.177	2.957
3-4	*gG'g'*	12.9	8371.48	3674.59	2800.99	-2.26	-0.70	1.19	2.65	0.767	2.889	4.812	0.166	2.849
3-5	*g'Gt*	13.0	6672.31	4213.21	3407.20	2.37	-0.2	0.62	2.46	1.801	-3.281	5.557	0.254	0.892
3-6	*g'Gg*	13.4	6659.25	4192.66	3407.76	0.98	0.80	1.91	2.30	1.831	-3.844	5.855	0.249	1.196
3-7	*tGg'*	13.4	6654.06	4217.74	3424.70	-2.00	-1.01	1.00	2.45	1.741	-4.859	7.476	0.244	1.611
3-8	*gGg'*	13.6	6647.61	4160.07	3369.64	-0.35	-2.5	0.35	2.54	1.745	-5.054	7.607	0.247	2.066
3-9	*tTt*	14.6	8044.65	3702.27	2776.74	-0.08	0.23	0.14	0.28	0.848	-0.917	5.915	0.238	4.035
3-10	*gTt*	14.7	7982.02	3632.68	2751.55	0.92	0.58	-1.6	1.93	0.798	-0.539	5.514	0.209	3.627
3-11	*tTg*	14.7	8107.53	3645.38	2761.19	-1.31	-1.56	0.09	2.04	0.804	-0.982	5.961	0.217	3.916
3-12	*g'Tg*	14.8	8063.43	3604.73	2724.93	-0.02	0.21	0.09	0.23	0.777	-0.798	5.738	0.2	3.68
3-13	*tTg'*	14.7	8053.64	3659.64	2766.13	-0.97	-0.19	1.63	1.90	0.801	0.246	4.883	0.213	3.338
3-14	*gTg'*	14.8	7980.46	3586.32	2739.23	0.02	0.24	-0.05	0.24	0.768	0.35	4.772	0.193	3.088
3-15	*gTg*	14.9	8057.56	3589.44	2738.06	-0.33	-1.28	-1.78	2.21	0.764	-0.391	5.238	0.191	3.399
3-16	*g'Tt*	14.9	7980.58	3659.33	2742.76	1.21	1.97	0.19	2.32	0.816	-1.07	5.968	0.216	3.857
3-17	*g'Tg'*	15.2	7965.29	3610.31	2728.92	0.28	1.66	1.89	2.53	0.793	0.048	4.962	0.205	3.263

3-18	*tG't*	15.6	8290.39	3581.39	2769.90	-0.91	-1.40	0.15	1.68	0.739	1.929	6.76	0.179	3.658
3-19	*g'G't*	16.0	8324.83	3562.32	2752.13	2.29	-1.34	2.00	3.32	0.797	0.763	7.049	0.193	3.584
3-20	*tGg*	16.1	6781.03	4094.07	3320.84	1.73	-0.44	2.51	3.08	1.742	-9.535	11.01	0.213	2.862
3-21	*gGt*	16.1	6733.24	4071.53	3254.39	-1.77	2.45	1.25	3.27	1.69	-6.373	7.862	0.228	3.019
3-22	*tG'g'*	16.3	8246.55	3590.18	2759.58	-0.01	-2.6	1.84	3.18	0.762	-0.832	8.191	0.182	4.226
4-1	*I*	15.4	8420.23	3949.17	2974.23	0.05	-0.35	-0.10	0.37	0.719	1.242	6.353	0.166	3.244
4-2	*II*	17.0	6939.42	4394.95	3490.83	0.20	0.19	-0.01	0.27	1.5	-4.823	6.559	0.226	2.576
4-3	*III*	17.1	8545.40	3955.87	2961.96	-1.16	0.58	1.80	2.21	0.796	0.525	7.181	0.196	3.887
4-4	*IV*	17.8	8218.32	3928.51	2935.77	1.56	0.49	1.42	2.17	0.883	-6.343	11.279	0.261	5.739
4-5	*V*	18.7	8140.71	3918.37	2946.97	1.00	1.82	-0.13	2.08	1.022	-12.142	17.565	0.327	7.906
4-6	*VI*	19.4	6912.63	4337.47	3472.61	-0.23	1.60	-1.98	2.55	1.428	-14.776	17.356	0.225	2.833
5-1	*tGG'g*	16.6	7712.67	3964.72	2894.25	3.05	1.56	1.14	3.61	2.244	0.922	1.032	0.709	4.041
5-2	*gGG'g*	16.8	7581.42	3960.16	2888.07	1.82	2.12	-0.65	2.87	2.379	2.624	-1.047	0.756	3.594
5-3	*tGGt*	17.6	7808.82	3654.28	3078.10	-2.34	0.37	-0.37	2.40	10.912	48.552	-36.809	3.961	15.911
5-4	*tGGg'*	17.8	9055.42	3045.70	2867.08	0	-0.4	0	0.40	4.638	74.521	-27.394	1.119	15.046
5-5	*gGGg'*	18.0	7781.62	3594.40	3037.78	-1.57	1.72	1.16	2.60	13.125	68.91	-49.989	4.686	17.661
5-6	*tGGg*	18.0	9172.40	2976.86	2811.89	0.18	1.37	1.35	1.93	3.989	75.947	-25.902	0.883	12.049
5-7	*tTGt*	18.4	13504.36	2461.04	2253.01	1.39	-0.23	1.53	2.08	0.806	57.805	-5.271	0.15	5.441
5-8	*gTGt*	18.4	13268.58	2429.85	2245.24	2.26	0.62	-0.08	2.35	0.809	63.75	-5.665	0.138	3.576
5-9	*tTGg*	18.5	13243.97	2417.81	2235.88	-0.06	0.04	-0.06	0.09	0.806	73.996	-6.657	0.12	3.799
5-10	*gGGg*	18.6	9275.26	2917.31	2760.34	0	3.27	0	3.27	3.355	72.075	-23.077	0.676	8.739
5-11	*g'TGt*	18.7	13266.36	2452.87	2244.27	1.88	2.19	1.59	3.30	0.824	58.564	-6.243	0.154	4.327
5-12	*g'TGg*	18.8	13035.78	2413.86	2224.87	0.43	2.37	-0.23	2.42	0.818	68.323	-7.286	0.134	2.987
5-13	*gTGg*	18.9	13137.66	2385.05	2219.49	0.99	0.79	-1.89	2.27	0.798	71.145	-6.679	0.123	2.049
5-14	*gTGg'*	19.2	13316.42	2372.25	2199.75	0.18	-0.02	0.15	0.23	0.757	67.694	-6.126	0.119	3.466
5-15	*tTTg*	19.2	20735.84	2008.65	1903.71	1.35	1.43	1.1	2.25	0.196	90.678	-2.357	0.017	0.516
5-16	*gTTg*	19.2	20618.92	1984.09	1889.10	0	0.13	0	0.13	0.195	90.861	-2.28	0.016	0.546
5-17	*g'GGg'*	19.2	9222.72	2904.02	2729.18	0	0.06	0	0.06	3.745	73.019	-24.046	0.875	13.935
5-18	*tTGg'*	19.2	13571.70	2390.35	2204.15	-0.85	-1.04	1.9	2.33	0.76	67.598	-5.876	0.129	5.406
5-19	*tTTt*	19.3	20905.33	2030.54	1917.15	0	3.12	0	3.12	0.194	89.844	-2.351	0.017	0.429
5-20	*g'TGg'*	19.3	13341.43	2386.14	2194.74	-0.27	1.38	1.8	2.29	0.779	65.175	-6.711	0.138	4.106
5-21	*gTTg'*	19.4	6781.03	4094.07	3320.84	1.73	-0.44	2.51	3.08	1.742	-9.535	11.01	0.213	2.862
5-22	*tGG't*	21.6	8246.55	3590.18	2759.58	-0.01	-2.6	1.84	3.18	0.762	-0.832	8.191	0.182	4.226
6-1	*gGT*	18.3	14447.39	2600.22	2436.83	-0.37	0.12	-0.14	0.41	0.918	165.267	-12.743	0.117	-4.064
6-2	*gGG*	19.3	11463.08	2818.59	2785.62	-0.43	-0.12	0.07	0.46	3.167	251.541	-44.648	-0.587	-36.494
6-3	*gGG'*	19.5	8614.15	3611.14	2861.42	0.30	-0.1	-0.12	0.34	5.572	60.711	-21.296	2.161	11.765
6-4	*g'GT*	20.1	14917.32	2587.13	2399.54	0.84	1.02	2.04	2.43	0.832	111.945	-9.14	0.143	4.166
6-5	*g'TT*	20.6	20342.02	2279.61	2143.03	-1.39	1.14	1.22	2.17	0.268	113.972	-3.833	0.031	0.75
6-6	*gG'G*	21.6	8738.87	3541.9	2834.76	-0.85	0.11	2.26	2.42	4.391	45.683	-18.92	1.465	6.618

6-7	*g'TG*	21.6	14894.25	2591.71	2390.48	-1.71	-0.21	1.62	2.36	0.891	108.23	-10.469	0.206	6.683
6-8	*gG'G'*	21.7	9590.85	3147.7	2937.21	1.63	-1.92	0.61	2.6	28.016	774.07	-245.319	9.017	130.69
6-9	*gG'G*	21.8	8448.97	3699.4	2856.52	-2.12	0.71	1.63	2.77	3.828	27.258	-12.729	1.303	5.894
6-10	*gTG*	22.1	14789.68	2561.25	2390.51	-1.86	1.15	-0.17	2.2	0.965	140.56	-6.004	0.236	5.663
7-1	*tGg'*	26.0	12972.96	2777.66	2496.08	2.16	1.22	0.13	2.49	1.399	80.853	-9.343	0.269	4.309
7-2	*gGg'*	27.4	9545.01	3221.38	2989.70	-1.80	0.88	1.41	2.45	7.222	158.198	-53.14	1.559	-0.154
7-3	*tTt*	28.3	22159.29	2186.01	2067.36	0.05	-0.31	0	0.32	0.223	97.884	-2.437	0.019	0.217
7-4	*tTg*	28.4	21995.82	2157.87	2052.10	-1.21	-1.13	-1.18	2.03	0.223	100.687	-2.727	0.019	0.21
7-5	*tGt*	28.6	13871.32	2603.65	2421.28	-0.19	1.54	-0.17	1.56	1.242	146.066	-14.926	0.223	3.241
7-6	*g'Gt*	28.8	8497.66	3653.29	2835.63	1.71	-0.11	0.06	1.71	2.959	13.75	0.692	1.005	6.9
7-7	*tGg*	29.1	13865.16	2580.14	2393.90	1.15	1.88	1.87	2.89	1.129	112.85	-11.371	0.206	3.676
7-8	*g'Gg*	29.4	8296.73	3666.79	2828.67	-0.75	0.40	1.81	2.00	2.996	5.627	2.542	0.98	7.643
7-9	*gTg'*	29.6	14803.27	2440.46	2302.86	-0.40	0.16	-0.02	0.43	0.903	126.854	-8.754	0.219	17.462
7-10	*gTt*	29.6	14873.79	2442.12	2330.85	0.23	-1.80	-0.37	1.85	0.833	158.099	-9.426	0.127	16.619
7-11	*gTg*	29.8	14712.03	2423.62	2310.93	-0.90	-1.62	1.54	2.41	0.795	130.246	-8.046	0.151	10.29
7-12	*gGt*	30.1	9500.41	3159.31	2940.16	0.71	-0.02	2.8	2.88	12.617	285.543	-99.035	3.714	48.092
8-1	*gg*	30.8	10039.67	3365.98	3144.31	0	-0.28	0	0.28	4.747	124.144	-40.497	1.072	8.163
8-2	*tg´*	33.4	14351.45	2822.56	2587.07	-1.00	-0.33	1.60	1.91	1.224	164.626	-15.771	0.237	2.368
8-3	*gg´*	34.3	9687.97	3521.79	2898.81	-0.82	1.50	1.62	2.36	3.612	70.841	-20.46	1.254	5.595
8-4	tt	36.2	23641.05	2354.68	2232.53	0	2.57	0	2.57	0.253	118.043	-3.438	0.024	-0.36
9-1	*I*	66.5	7919.84	3889.44	2869.30	-0.74	-0.35	1.42	1.64	0.769	0.592	5.551	0.195	3.387
9-2	*II*	66.6	7881.98	3873.15	2870.48	-0.92	1.35	0.52	1.72	0.753	0.343	5.972	0.186	3.367
9-3	*III*	67.3	6414.71	4385.18	3448.30	-1.06	1.3	-0.34	1.71	1.626	-4.973	6.264	0.274	1.922
10-1	*I*	70.7	8902.91	3180.95	2964.36	-0.25	1.29	0.6	1.44	6.168	107.08	-40.273	1.625	18.712
10-2	*II*	70.9	8952.7	3126.35	2925.55	1.63	-0.46	0.48	1.76	5.174	96.514	-34.543	1.289	15.329
10-3	*III*	71.0	12897.53	2602.48	2380.02	0.39	1.53	-0.58	1.68	1.088	86.862	-9.557	0.216	4.15
10-4	*IV*	71.0	13403.6	2510.84	2320.02	0.53	0.41	1.32	1.48	0.777	80.912	-6.78	0.117	2.129
10-5	*V*	71.0	13005.74	2601.24	2366.43	-1.01	-0.16	1.34	1.69	1.06	80.589	-8.418	0.218	5.041
10-6	*VI*	71.0	20726.98	2126.42	2011.45	1.03	-0.45	1.33	1.74	0.236	111.063	-3.004	0.022	-0.009
10-7	*VII*	71.1	13345.57	2494.63	2312.65	1.54	-0.74	-0.79	1.88	0.742	73.95	-6.029	0.108	3.154
10-8	*VIII*	72.0	7798.05	3791.33	2827.87	-0.04	0.7	1.3	1.48	2.891	1.646	2.156	0.93	6.916
10-9	*IX*	72.5	7775.17	3717.08	2801.32	1.48	0.16	-0.44	1.55	3.061	-2.705	6.814	0.964	7.569
11-1	I	77.2	16712.65	2479.57	2428.62	0.3	0.75	1.43	1.64	2.81	966.856	-76.966	-0.404	-50.736
11-2	II	77.3	9959.25	3291.68	2844.13	0.46	1.18	-0.3	1.30	10.747	336.597	-104.531	3.637	16.722
11-3	III	77.3	13075.14	2883.4	2675.06	-0.06	0.33	1.48	1.51	5.864	694.644	-117.036	-0.87	38.874

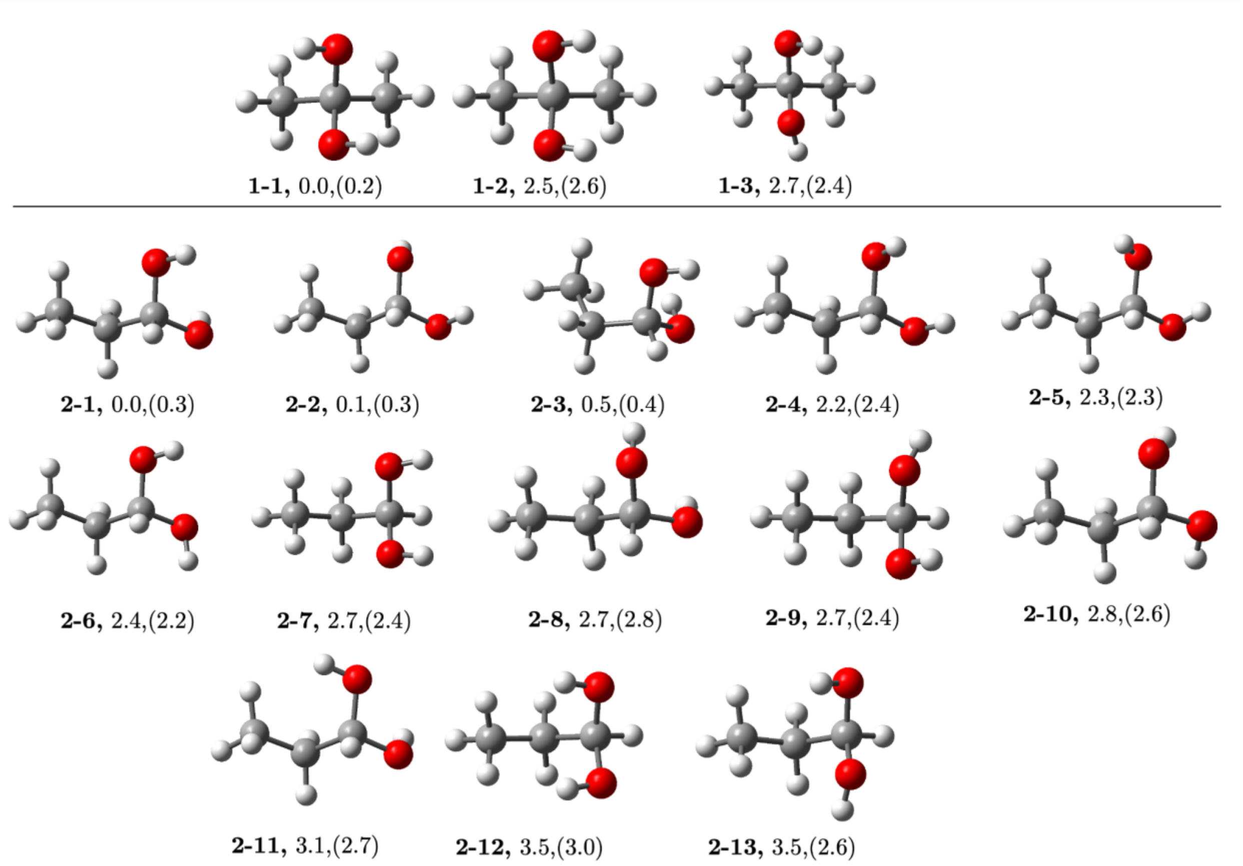

Figure S1. All conformers of the geminal diols **1** and **2**. Relative energy (kcal/mol) obtained at the CCSD(T)/aug-cc-pVTZ//MP2/aug-cc-pvTZ level of theory. The value in parenthesis represents the dipole moment (Debye).

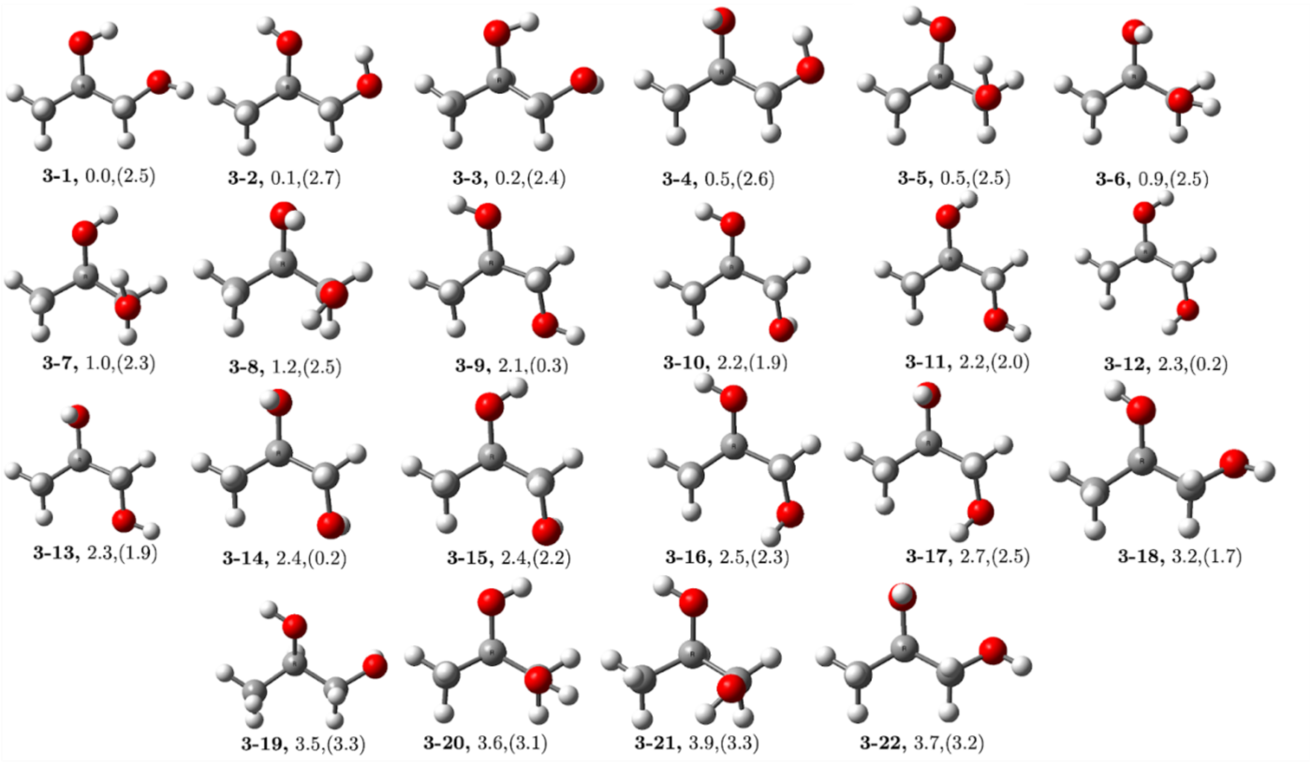

Figure S2. All conformers of isomer **3**. Relative energy (kcal/mol) obtained at the CCSD(T)/aug-cc-pVTZ//MP2/aug-cc-pvTZ level of theory. The value in parenthesis represents the dipole moment (Debyes).

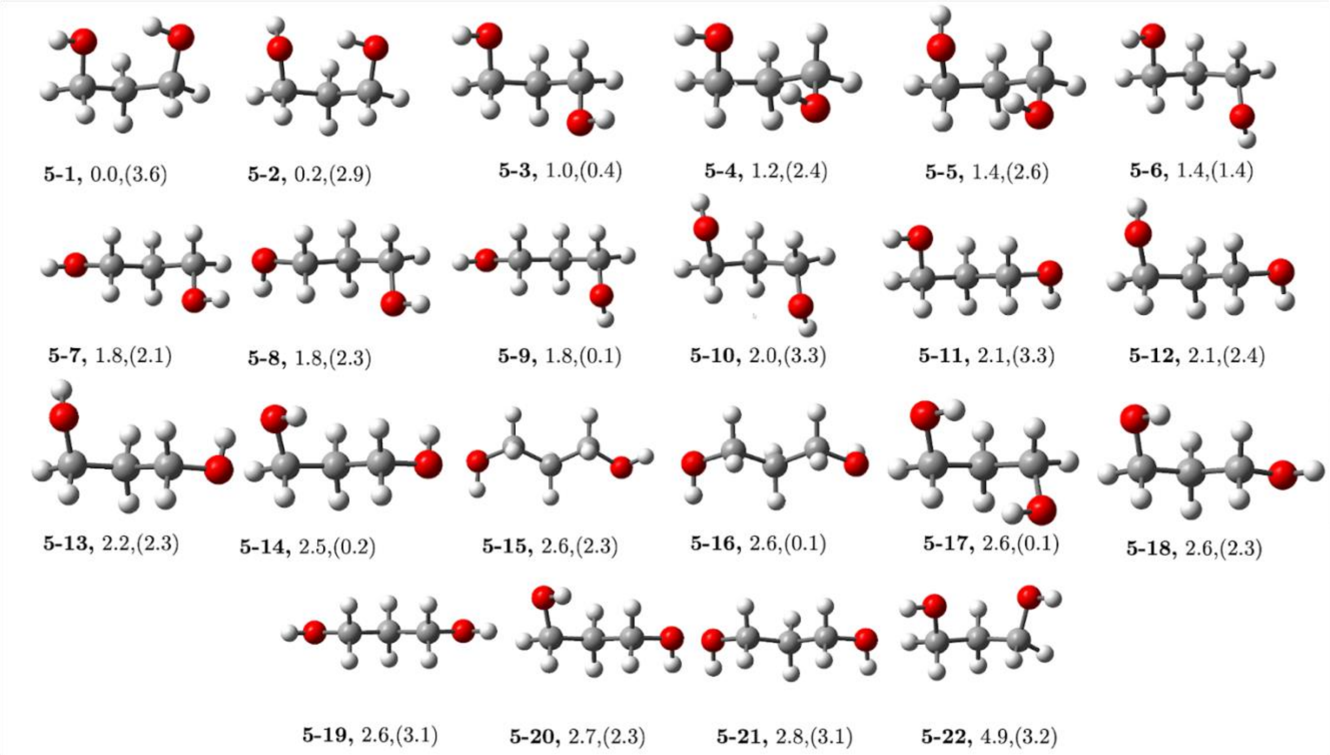

Figure S3. All conformers of isomer **5**. Relative energy (kcal/mol) obtained at the CCSD(T)/aug-cc-pVTZ//MP2/aug-cc-pvTZ level of theory. The value in parenthesis represents the dipole moment (Debye).

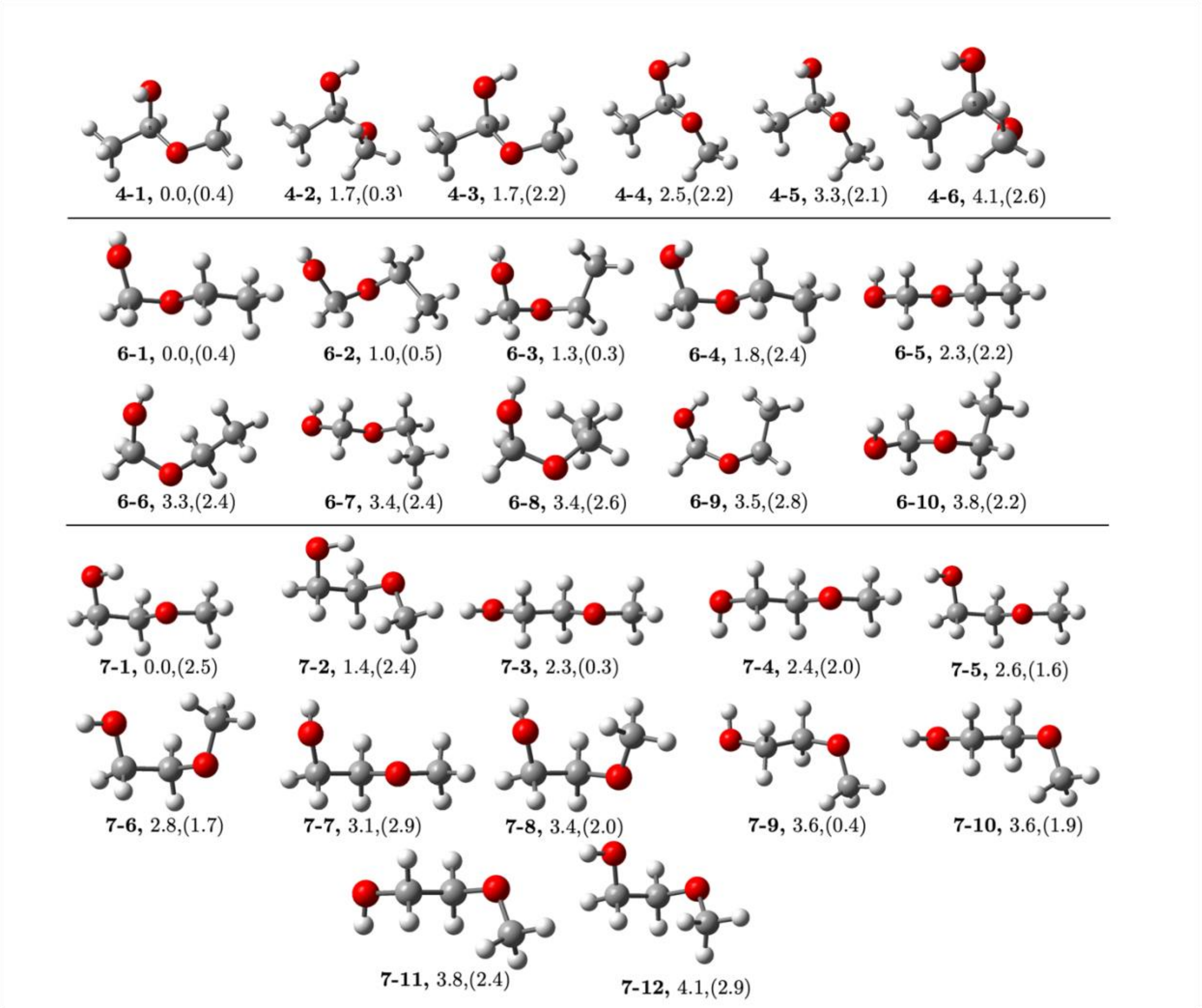

Figure S4. Conformers of the hydroxy ethers **4**, **6,** and **7**. Relative energy (kcal/mol) obtained at the CCSD(T)/aug-cc-pVTZ//MP2/aug-cc-pvTZ level of theory. The value in parenthesis represents the dipole moment (Debye).

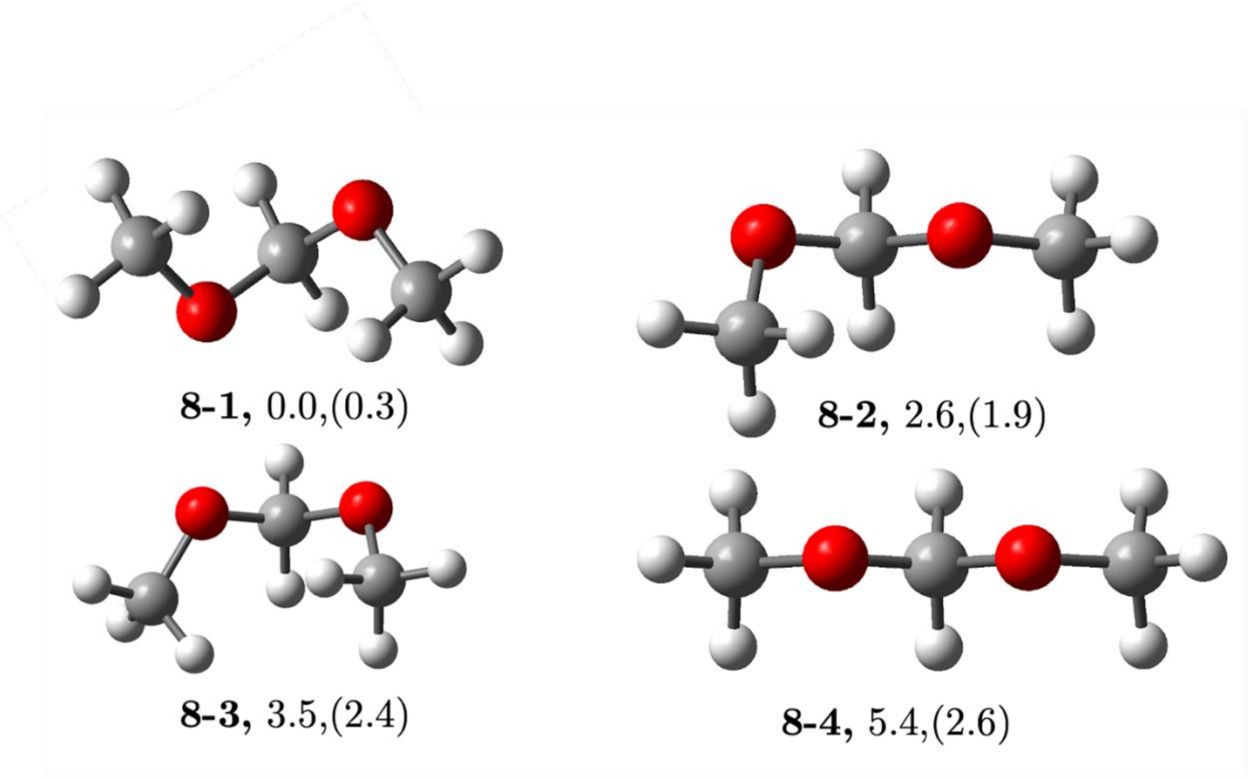

Figure S5. Conformers of the diether **8**. Relative energy (kcal/mol) obtained at the CCSD(T)/aug-cc-pVTZ//MP2/aug-cc-pvTZ level of theory. The value in parenthesis represents the dipole moment (Debye).

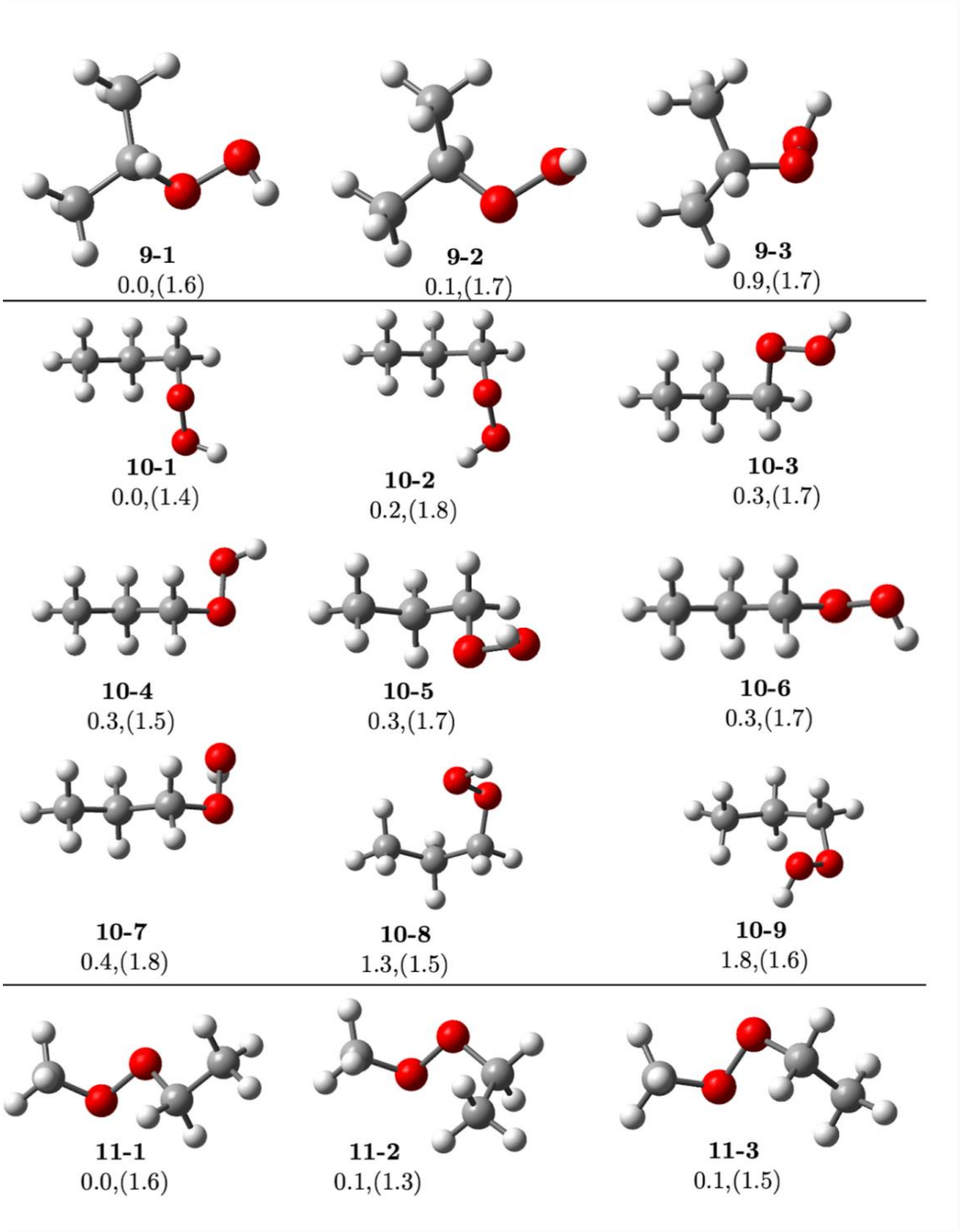

Figure S6. Conformers of the peroxides **9**, **10**, and **11** identified in the PES exploration. Relative energy (kcal/mol) obtained at the CCSD(T)/aug-cc-pVTZ//MP2/aug-cc-pvTZ level of theory. The value in parenthesis represents the dipole moment (Debye).

Cartesian coordinates. Cartesian coordinates of transition states and product complexes involved in the uncatalyzed decomposition of **1** (TS1, PC1) and **2** (TS2, PC2). All the structures are optimized at MP2/aug-cc-pVTZ level of theory.

TS1

C	-0.554590000	1.438216000	-0.170125000
C	-0.158907000	0.021862000	0.167150000
C	-1.119312000	-1.048802000	-0.276983000
O	1.257162000	-0.326893000	-0.775942000
O	0.496277000	-0.175783000	1.292037000
H	-0.848707000	1.548951000	-1.212257000
H	0.256348000	2.120488000	0.078294000
H	-1.407546000	1.692738000	0.460580000
H	-1.435376000	-0.913955000	-1.309007000
H	-0.657090000	-2.023845000	-0.148805000
H	-1.992467000	-0.991084000	0.374636000
H	1.640120000	0.510344000	-1.081136000
H	1.414057000	-0.389888000	0.388680000

PC1

C	-0.303778000	1.352177000	-0.006761000
C	-0.729247000	-0.091016000	-0.006227000
C	-2.210053000	-0.359657000	0.016989000
O	2.734311000	-0.006629000	0.012695000
O	0.079302000	-1.010463000	-0.024732000
H	-0.638044000	1.817223000	-0.935777000
H	0.774991000	1.441195000	0.079360000
H	-0.795281000	1.882711000	0.809152000
H	-2.704088000	0.190330000	-0.784137000
H	-2.400656000	-1.423710000	-0.082653000
H	-2.625565000	0.002949000	0.958363000
H	3.431570000	-0.666806000	0.034589000
H	1.906633000	-0.516184000	-0.006609000

TS2

C	-2.096160000	0.067609000	0.034331000
C	-0.778433000	-0.670403000	-0.196020000
C	0.337821000	0.151412000	0.391524000
O	1.807855000	-0.626409000	0.073167000
O	0.704198000	1.256459000	-0.214648000
H	-2.285869000	0.199903000	1.099598000
H	-2.928913000	-0.490332000	-0.389791000
H	-2.057410000	1.050363000	-0.431231000
H	-0.803668000	-1.657535000	0.268927000
H	-0.597587000	-0.780739000	-1.267420000
H	0.366459000	0.135149000	1.488821000
H	1.668167000	-1.244248000	-0.662403000

H	1.763031000	0.455331000	-0.353657000

PC2

C	-1.423433000	-1.170970000	-0.405476000
C	-0.977351000	-0.156368000	0.654886000
C	-0.603826000	1.121656000	-0.026568000
O	2.105651000	-0.869982000	0.055751000
O	0.539762000	1.502495000	-0.218939000
H	-2.231023000	-0.772832000	-1.020575000
H	-1.780880000	-2.080519000	0.072420000
H	-0.584574000	-1.428225000	-1.049468000
H	-1.801813000	0.049330000	1.340574000
H	-0.124549000	-0.537662000	1.212255000
H	-1.445358000	1.725825000	-0.410904000
H	3.043341000	-0.825917000	-0.149067000
H	1.789220000	0.043985000	-0.026785000

Cartesian coordinates. Cartesian coordinates of the reactive complex, transition states and product complex involved in the water-catalyzed decomposition of **1** (RC1, TS1, PC1) and **2** (RC2, TS2, PC2). All the structures are optimized at MP2/aug-cc-pVTZ level of theory.

RC1

C	0.487152000	1.510711000	-0.032960000
C	0.604708000	-0.005621000	-0.034769000
C	2.038792000	-0.486769000	-0.006445000
O	-0.111648000	-0.539386000	1.110951000
O	0.005752000	-0.567555000	-1.159849000
H	0.943161000	1.936133000	0.862112000
H	-0.563147000	1.798142000	-0.066408000
H	0.994905000	1.915726000	-0.906324000
H	2.548810000	-0.113262000	0.881078000
H	2.046816000	-1.574038000	0.002892000
H	2.564775000	-0.130969000	-0.890339000
H	0.235755000	-0.111242000	1.902852000
H	-0.931082000	-0.306408000	-1.151505000
O	-2.569964000	0.029829000	-0.045804000
H	-1.934363000	-0.291506000	0.617924000
H	-3.282671000	-0.615608000	-0.049626000

TS1

C	0.720206000	1.501211000	0.156522000
C	0.583917000	0.033967000	-0.202507000
C	1.844469000	-0.781077000	-0.086373000
O	-0.324074000	-0.566292000	1.175422000
O	-0.168931000	-0.243555000	-1.221291000
H	1.235664000	1.652509000	1.104871000
H	-0.260802000	1.972886000	0.181289000
H	1.306313000	1.976114000	-0.630940000
H	2.340512000	-0.625499000	0.869629000
H	1.602743000	-1.832569000	-0.212964000
H	2.516931000	-0.474409000	-0.888476000
H	-0.168017000	0.007444000	1.937721000
H	-1.353542000	-0.027027000	-0.845971000
O	-2.241659000	0.038354000	-0.099076000
H	-1.376903000	-0.326786000	0.737081000
H	-2.857141000	-0.675327000	-0.298528000

PC1

C	-0.776716000	-0.311866000	1.445973000
C	-0.988607000	-0.416590000	-0.037547000
C	-2.068238000	0.442039000	-0.628134000
O	1.041505000	1.788941000	-0.071229000
O	-0.307197000	-1.154332000	-0.741520000
H	-0.702545000	0.736308000	1.729846000

H	0.122314000	-0.843020000	1.745054000
H	-1.642969000	-0.740626000	1.953597000
H	-1.684427000	1.463838000	-0.647575000
H	-2.295868000	0.121239000	-1.640137000
H	-2.963316000	0.431916000	-0.007607000
H	1.613887000	2.554739000	0.022687000
H	1.475669000	-1.131790000	-0.317687000
O	2.323085000	-0.739463000	-0.034036000
H	1.647615000	1.026440000	-0.079792000
H	2.971857000	-1.061706000	-0.665852000

RC2

C	2.546858000	-0.585209000	0.294499000
C	1.595391000	0.546714000	-0.068806000
C	0.150272000	0.181313000	0.181476000
O	-0.722122000	1.294005000	-0.042624000
O	-0.177547000	-0.892528000	-0.652160000
H	2.443157000	-0.853660000	1.346449000
H	3.581526000	-0.291027000	0.125879000
H	2.335556000	-1.468942000	-0.302978000
H	1.817418000	1.443184000	0.510324000
H	1.694112000	0.800026000	-1.127921000
H	-0.017110000	-0.067884000	1.232950000
H	-0.592253000	1.563575000	-0.961716000
H	-1.084185000	-1.158413000	-0.426662000
O	-2.860138000	-0.488487000	0.193458000
H	-2.409077000	0.372242000	0.216253000
H	-3.445812000	-0.499932000	0.955021000

TS2

C	-2.496804000	-0.469400000	0.238756000
C	-1.510561000	0.576207000	-0.261167000
C	-0.114565000	0.416778000	0.283051000
O	0.363183000	-1.142650000	-0.243812000
O	0.781352000	1.258115000	-0.125714000
H	-2.540493000	-0.469168000	1.328059000
H	-2.212156000	-1.469598000	-0.081840000
H	-3.498861000	-0.262926000	-0.133312000
H	-1.448180000	0.592607000	-1.351620000
H	-1.826608000	1.577222000	0.040775000
H	-0.094963000	0.130679000	1.345497000
H	0.241944000	-1.115803000	-1.205798000
H	1.489365000	-0.948845000	-0.079954000
O	2.573023000	-0.290957000	0.067236000
H	2.988115000	-0.400440000	0.929109000
H	1.892961000	0.628700000	0.063560000

PC2

C	-2.699442000	0.704090000	0.557637000
C	-2.093795000	-0.241423000	-0.483494000
C	-0.620014000	-0.361964000	-0.265239000
O	1.925316000	1.790240000	-0.258715000
O	-0.051608000	-1.419779000	-0.023897000
H	-2.207820000	1.676229000	0.532903000
H	-2.591450000	0.293788000	1.560558000
H	-3.759091000	0.856038000	0.363860000
H	-2.544850000	-1.231241000	-0.445464000
H	-2.244315000	0.177205000	-1.482187000
H	-0.041151000	0.574721000	-0.307631000
H	2.425542000	2.451849000	0.225603000
H	2.346637000	0.941496000	-0.029265000
O	2.644811000	-0.840008000	0.365803000
H	3.206406000	-1.395435000	-0.181383000
H	1.741438000	-1.192489000	0.244043000

Cartesian Coordinates. Cartesian coordinates of all the conformers identified. All the structures are optimized at MP2/aug-cc-pVTZ. The relative energy (ΔE, in kcal/mol) includes the zero-point energy correction.

Isomer 1

Conformer 1-1

ΔE= 0.00

C	-0.000000000	1.262271000	-0.824979000
C	0.000000000	0.000000000	0.012727000
C	-0.000000000	-1.262271000	-0.824979000
O	1.159115000	-0.081800000	0.826584000
O	-1.159115000	0.081800000	0.826584000
H	0.884496000	1.283601000	-1.457440000
H	0.003973000	2.134781000	-0.171752000
H	-0.892621000	1.302209000	-1.445911000
H	0.892621000	-1.302209000	-1.445911000
H	-0.003973000	-2.134781000	-0.171752000
H	-0.884496000	-1.283601000	-1.457440000
H	1.172324000	0.713481000	1.374122000
H	-1.172324000	-0.713481000	1.374122000

Conformer 1-2

ΔE= 2.55

C	-0.550110000	1.408255000	0.000000000
C	-0.027027000	-0.009252000	0.000000000
C	1.491042000	-0.081684000	0.000000000
O	-0.550110000	-0.617620000	1.170338000
O	-0.550110000	-0.617620000	-1.170338000
H	-1.637454000	1.381385000	0.000000000
H	-0.204757000	1.929837000	-0.889444000
H	-0.204757000	1.929837000	0.889444000
H	1.891440000	0.402495000	-0.888398000
H	1.891440000	0.402495000	0.888398000
H	1.817956000	-1.125243000	0.000000000
H	-0.117764000	-1.471398000	1.289405000
H	-0.117764000	-1.471398000	-1.289405000

Conformer 1-3

ΔE= 2.80

C	-1.126527000	0.971466000	-0.190251000
C	0.011532000	-0.022164000	-0.010975000
C	1.368413000	0.649452000	0.030848000
O	-0.204312000	-0.805961000	1.165522000
O	0.055789000	-0.938323000	-1.074916000
H	-1.157124000	1.682359000	0.636754000
H	-2.076362000	0.438619000	-0.229739000
H	-0.991071000	1.524441000	-1.117578000
H	1.557797000	1.172292000	-0.904980000

H	1.407781000	1.365375000	0.850789000
H	2.132012000	-0.110009000	0.180196000
H	-0.477928000	-0.214270000	1.875517000
H	-0.727440000	-1.497054000	-0.993546000

Isomer 2

Conformer 2-1

ΔE=0.00

C	*-2.010583000*	*0.047475000*	*0.102663000*
C	*-0.718484000*	*-0.671614000*	*-0.260068000*
C	*0.503860000*	*-0.002975000*	*0.329239000*
O	*1.691562000*	*-0.726112000*	*0.090318000*
O	*0.575812000*	*1.294124000*	*-0.226182000*
H	*-2.135281000*	*0.093950000*	*1.185142000*
H	*-2.873258000*	*-0.473393000*	*-0.309740000*
H	*-2.005085000*	*1.065001000*	*-0.281331000*
H	*-0.732903000*	*-1.700946000*	*0.098688000*
H	*-0.589889000*	*-0.699173000*	*-1.345675000*
H	*0.433270000*	*0.048271000*	*1.418922000*
H	*1.765051000*	*-0.838812000*	*-0.865991000*
H	*1.350354000*	*1.723694000*	*0.155889000*

Conformer 2-2

ΔE=0.11

C	-2.011249000	0.045474000	0.116355000
C	-0.722546000	-0.657953000	-0.284244000
C	0.499051000	-0.006136000	0.325636000
O	1.612763000	-0.789548000	-0.047367000
O	0.665501000	1.337509000	-0.075348000
H	-2.146392000	0.020098000	1.197994000
H	-2.874092000	-0.437659000	-0.338881000
H	-1.998260000	1.088665000	-0.193530000
H	-0.736015000	-1.702302000	0.028799000
H	-0.594092000	-0.652794000	-1.370059000
H	0.409234000	0.050131000	1.413642000
H	2.393404000	-0.372824000	0.336648000
H	0.728566000	1.334688000	-1.039368000

Conformer 2-3

ΔE=0.54

C	1.719666000	0.009722000	-0.493252000
C	0.843912000	-0.065250000	0.751903000
C	-0.640744000	-0.006581000	0.446106000
O	-1.034246000	1.185679000	-0.198450000
O	-0.948188000	-1.137813000	-0.344129000
H	1.473918000	-0.788484000	-1.190642000
H	2.770169000	-0.083943000	-0.223373000
H	1.610186000	0.966624000	-1.005960000

H	1.032833000	-0.997340000	1.285538000
H	1.073134000	0.756908000	1.430322000
H	-1.222490000	-0.014396000	1.369870000
H	-0.523347000	1.247339000	-1.015049000
H	-1.891937000	-1.096983000	-0.538623000

Conformer 2-4

ΔE=2.18

C	-2.006525000	0.043608000	0.130578000
C	-0.721636000	-0.663802000	-0.273663000
C	0.504834000	0.001112000	0.301440000
O	1.610141000	-0.809334000	-0.037578000
O	0.603941000	1.295018000	-0.263120000
H	-2.123414000	0.052736000	1.215201000
H	-2.874036000	-0.460691000	-0.291858000
H	-2.004711000	1.073697000	-0.219498000
H	-0.723741000	-1.700772000	0.061415000
H	-0.602110000	-0.667745000	-1.358153000
H	0.428583000	0.089235000	1.391988000
H	2.406755000	-0.412453000	0.331011000
H	1.119971000	1.855013000	0.325348000

Conformer 2-5

ΔE=2.33

C	-2.001684000	0.047300000	0.094878000
C	-0.719219000	-0.694901000	-0.251613000
C	0.504249000	-0.009858000	0.313309000
O	1.616405000	-0.788425000	-0.022544000
O	0.702306000	1.282378000	-0.252448000
H	-2.124933000	0.138356000	1.175492000
H	-2.872521000	-0.478402000	-0.292444000
H	-2.001160000	1.047949000	-0.336161000
H	-0.743955000	-1.712207000	0.141098000
H	-0.582838000	-0.756975000	-1.331751000
H	0.416539000	0.095612000	1.403589000
H	2.400286000	-0.269792000	0.193934000
H	0.258822000	1.928593000	0.306730000

Conformer 2-6

ΔE=2.44

C	-2.015466000	0.034001000	0.120475000
C	-0.724321000	-0.657437000	-0.295947000
C	0.497544000	-0.001821000	0.307305000
O	1.704446000	-0.651009000	-0.077657000
O	0.566782000	1.305556000	-0.182901000
H	-2.125408000	0.034554000	1.205830000
H	-2.881468000	-0.473420000	-0.301561000
H	-2.021956000	1.067117000	-0.219910000
H	-0.732216000	-1.703456000	0.017886000

H	-0.597030000	-0.638356000	-1.378915000
H	0.429596000	0.001519000	1.404496000
H	1.801799000	-1.445676000	0.458761000
H	1.410317000	1.672876000	0.106875000

Conformer 2-7

ΔE=2.61

C	1.717306000	0.003383000	-0.503489000
C	0.846948000	-0.004021000	0.747275000
C	-0.631215000	-0.001244000	0.428790000
O	-0.909747000	1.173068000	-0.309540000
O	-0.910386000	-1.175446000	-0.308627000
H	1.520534000	-0.874894000	-1.114441000
H	2.771120000	0.001738000	-0.228764000
H	1.520684000	0.888992000	-1.103881000
H	1.051586000	-0.888672000	1.350852000
H	1.052414000	0.872894000	1.361727000
H	-1.225576000	0.000064000	1.348755000
H	-1.862655000	1.308984000	-0.327881000
H	-1.865275000	-1.278788000	-0.376482000

Conformer 2-8

ΔE=2.85

C	-2.003744000	0.010895000	0.099906000
C	-0.692642000	-0.671960000	-0.263297000
C	0.517541000	0.022047000	0.337905000
O	1.703739000	-0.701274000	0.088806000
O	0.619134000	1.365538000	-0.082547000
H	-2.143268000	0.018979000	1.180988000
H	-2.850386000	-0.510902000	-0.343220000
H	-2.012961000	1.042385000	-0.245474000
H	-0.691512000	-1.707787000	0.079158000
H	-0.564526000	-0.691240000	-1.352573000
H	0.432710000	0.083959000	1.421829000
H	1.643546000	-1.088560000	-0.792077000
H	0.676483000	1.373170000	-1.045787000

Conformer 2-9

ΔE=2.76

C	1.720826000	0.007419000	-0.512580000
C	0.859015000	0.064250000	0.741044000
C	-0.625178000	-0.004610000	0.439703000
O	-1.055300000	1.046384000	-0.421880000
O	-0.874349000	-1.209044000	-0.226115000
H	1.574809000	-0.934913000	-1.035386000
H	2.774766000	0.098185000	-0.254004000
H	1.462683000	0.816920000	-1.192560000
H	1.099152000	-0.761318000	1.413633000
H	1.040973000	0.990418000	1.290751000

H	-1.204091000	0.048102000	1.370670000
H	-1.253571000	1.816195000	0.122281000
H	-1.785509000	-1.174661000	-0.540435000

Conformer 2-10

ΔE=2.91

C	-2.009382000	0.040223000	0.092887000
C	-0.709854000	-0.660243000	-0.277678000
C	0.499794000	0.020189000	0.341796000
O	1.702918000	-0.659290000	0.014334000
O	0.644707000	1.348054000	-0.080123000
H	-2.156901000	0.034832000	1.173068000
H	-2.864246000	-0.454664000	-0.364791000
H	-1.990107000	1.076354000	-0.238237000
H	-0.736052000	-1.700185000	0.061120000
H	-0.570126000	-0.671114000	-1.362009000
H	0.387268000	0.081946000	1.427712000
H	1.497033000	-1.590076000	-0.123330000
H	0.968782000	1.311774000	-0.989255000

Conformer 2-11

ΔE=3.23

C	-2.004748000	0.005826000	0.087387000
C	-0.694280000	-0.688322000	-0.264172000
C	0.522012000	-0.007689000	0.341037000
O	1.705196000	-0.701177000	0.075171000
O	0.695421000	1.309714000	-0.163606000
H	-2.114147000	0.109375000	1.167794000
H	-2.857315000	-0.562899000	-0.279478000
H	-2.073911000	0.999531000	-0.358311000
H	-0.703266000	-1.714849000	0.103686000
H	-0.556541000	-0.726069000	-1.347662000
H	0.440825000	0.013937000	1.432113000
H	1.875402000	-0.609731000	-0.870854000
H	-0.153886000	1.763518000	-0.125310000

Conformer 2-12

ΔE=3.65

C	1.730507000	0.000140000	-0.452082000
C	0.811193000	-0.000508000	0.765501000
C	-0.673511000	-0.000027000	0.421743000
O	-1.061065000	1.172823000	-0.261687000
O	-1.061670000	-1.172339000	-0.262290000
H	1.590713000	-0.883735000	-1.076404000
H	2.772518000	-0.000727000	-0.137480000
H	1.591528000	0.885388000	-1.074606000
H	1.009309000	-0.881242000	1.376936000
H	1.009624000	0.879460000	1.377932000
H	-1.271208000	-0.000044000	1.330137000

H	-0.465178000	1.302272000	-1.008121000
H	-0.464558000	-1.302869000	-1.007554000

Conformer 2-13

Δ*E*=3.66

C	-1.716442000	-0.050952000	-0.488325000
C	-0.837182000	0.099918000	0.746333000
C	0.653848000	0.009168000	0.445594000
O	1.015617000	-1.197969000	-0.158873000
O	1.076878000	1.047811000	-0.430394000
H	-1.469464000	0.691816000	-1.245456000
H	-2.766589000	0.068257000	-0.227588000
H	-1.602818000	-1.041138000	-0.929036000
H	-1.029252000	1.058835000	1.236549000
H	-1.070319000	-0.677843000	1.475022000
H	1.226810000	0.044613000	1.375151000
H	0.682241000	-1.168276000	-1.064121000
H	0.688087000	1.876198000	-0.128000000

Isomer 3

Conformer 3-1

Δ*E*=0.00

C	-1.780634000	-0.586329000	0.030373000
C	-0.461374000	0.049967000	-0.342817000
O	-0.457705000	1.372209000	0.183543000
C	0.711155000	-0.727102000	0.213380000
O	1.889617000	-0.011480000	-0.165980000
H	-1.878878000	-0.634264000	1.114530000
H	-1.853610000	-1.593435000	-0.379218000
H	-2.602151000	0.009322000	-0.362011000
H	-0.356549000	0.086701000	-1.433528000
H	0.445336000	1.699078000	0.076129000
H	0.620286000	-0.776878000	1.301912000
H	0.720262000	-1.739994000	-0.198711000
H	2.635125000	-0.355578000	0.334774000

Conformer 3-2

Δ*E*=0.21

C	-1.760660000	-0.618924000	0.028061000
C	-0.443759000	0.033283000	-0.342497000
O	-0.332235000	1.350491000	0.216053000
C	0.739593000	-0.722279000	0.222237000
O	1.969396000	-0.116751000	-0.136689000
H	-1.856264000	-0.678131000	1.111721000
H	-1.821901000	-1.624587000	-0.388939000
H	-2.600704000	-0.042308000	-0.359212000
H	-0.331545000	0.088213000	-1.429801000
H	-0.957996000	1.927607000	-0.233841000

H	0.629683000	-0.775982000	1.311241000
H	0.756644000	-1.735601000	-0.176523000
H	1.873745000	0.818384000	0.083638000

Conformer 3-3

ΔE=0.33

C	1.785345000	-0.577805000	-0.019800000
C	0.458541000	0.048015000	0.345545000
O	0.451899000	1.378469000	-0.156026000
C	-0.708073000	-0.723242000	-0.240411000
O	-1.943672000	-0.046308000	0.003629000
H	1.890318000	-0.618411000	-1.103581000
H	1.860895000	-1.587409000	0.383750000
H	2.601443000	0.019413000	0.381784000
H	0.348630000	0.067262000	1.439179000
H	-0.472687000	1.661862000	-0.150226000
H	-0.603506000	-0.762213000	-1.324283000
H	-0.730179000	-1.743759000	0.150976000
H	-2.175607000	-0.175846000	0.929567000

Conformer 3-4

ΔE=0.66

C	-1.766549000	-0.595431000	0.019692000
C	-0.438069000	0.027419000	-0.355025000
O	-0.358900000	1.400864000	0.054464000
C	0.737449000	-0.730311000	0.233201000
O	1.968572000	-0.120544000	-0.109202000
H	-1.884438000	-0.618759000	1.105334000
H	-1.829552000	-1.619804000	-0.347256000
H	-2.587916000	-0.022260000	-0.405751000
H	-0.322369000	0.063238000	-1.438779000
H	-0.655847000	1.447501000	0.971741000
H	0.615814000	-0.783517000	1.323701000
H	0.764254000	-1.748412000	-0.155233000
H	1.825689000	0.829384000	-0.003057000

Conformer 3-5

ΔE=0.61

C	1.215848000	-1.045574000	-0.269433000
C	0.541594000	0.075382000	0.496449000
O	0.651896000	1.325687000	-0.198104000
C	-0.951342000	-0.140214000	0.661170000
O	-1.613631000	-0.214358000	-0.591003000
H	0.738994000	-1.177513000	-1.238418000
H	1.143679000	-1.980239000	0.286831000
H	2.274400000	-0.827336000	-0.419279000
H	0.984567000	0.169522000	1.494247000
H	1.558572000	1.420986000	-0.508090000
H	-1.134778000	-1.084287000	1.172460000

H	-1.359422000	0.671924000	1.269801000
H	-1.348727000	0.578751000	-1.073810000

Conformer 3-6

ΔE=1.00

C	1.246448000	-1.028376000	-0.264380000
C	0.570874000	0.114929000	0.474688000
O	0.751571000	1.351855000	-0.208376000
C	-0.908979000	-0.138064000	0.690472000
O	-1.507730000	-0.204863000	-0.607384000
H	0.775672000	-1.180548000	-1.234608000
H	1.170936000	-1.955390000	0.305339000
H	2.298061000	-0.794835000	-0.416773000
H	1.035750000	0.250088000	1.452744000
H	0.167722000	1.313648000	-0.977482000
H	-1.057090000	-1.074515000	1.235561000
H	-1.331969000	0.687544000	1.266247000
H	-2.459868000	-0.112862000	-0.509623000

Conformer 3-7

ΔE=1.07

C	1.227601000	-1.048553000	-0.238297000
C	0.548506000	0.094770000	0.481668000
O	0.745891000	1.264755000	-0.330003000
C	-0.944227000	-0.129120000	0.669198000
O	-1.613866000	-0.250820000	-0.573885000
H	0.768064000	-1.202622000	-1.212308000
H	1.129978000	-1.966359000	0.340390000
H	2.284021000	-0.828659000	-0.376395000
H	1.006962000	0.242257000	1.465217000
H	0.509731000	2.037623000	0.195917000
H	-1.121120000	-1.052939000	1.220026000
H	-1.362048000	0.699961000	1.253740000
H	-1.263071000	0.456677000	-1.130902000

Conformer 3-8

ΔE=1.34

C	1.252185000	-1.030684000	-0.232913000
C	0.570108000	0.131334000	0.471778000
O	0.750434000	1.343332000	-0.252415000
C	-0.914220000	-0.111201000	0.694724000
O	-1.622827000	-0.132222000	-0.547105000
H	0.811794000	-1.197639000	-1.217215000
H	1.167903000	-1.948293000	0.351405000
H	2.306333000	-0.805158000	-0.378811000
H	1.037746000	0.291073000	1.445590000
H	0.084173000	1.331824000	-0.953266000
H	-1.074685000	-1.033986000	1.258260000
H	-1.334842000	0.721202000	1.255254000

H	-1.467721000	-0.984592000	-0.966589000

Conformer 3-9

Δ*E*=2.15

C	0.723898000	1.367683000	0.147163000
C	0.462981000	-0.043280000	-0.338100000
O	1.502871000	-0.942986000	0.050683000
C	-0.795703000	-0.628498000	0.267122000
O	-1.890578000	0.164783000	-0.183844000
H	0.845138000	1.368974000	1.230645000
H	-0.109068000	2.016269000	-0.118528000
H	1.631322000	1.769937000	-0.305948000
H	0.347740000	-0.046581000	-1.427022000
H	2.342105000	-0.562812000	-0.229171000
H	-0.893238000	-1.666975000	-0.056819000
H	-0.707518000	-0.606130000	1.357370000
H	-2.701878000	-0.222486000	0.157640000

Conformer 3-10

Δ*E*=2.40

C	0.758000000	1.364494000	0.127085000
C	0.455072000	-0.045873000	-0.333732000
O	1.487954000	-0.962914000	0.029160000
C	-0.803655000	-0.597314000	0.314800000
O	-1.961680000	0.141350000	-0.058641000
H	0.933742000	1.368599000	1.203015000
H	-0.082293000	2.020262000	-0.095521000
H	1.643904000	1.757821000	-0.373856000
H	0.314496000	-0.056398000	-1.422549000
H	2.332599000	-0.577438000	-0.226703000
H	-0.900931000	-1.656267000	0.065865000
H	-0.716254000	-0.508482000	1.397072000
H	-2.191962000	-0.103428000	-0.960393000

Conformer 3-11

Δ*E*=2.32

C	0.733659000	1.379971000	0.123398000
C	0.467704000	-0.034994000	-0.332777000
O	1.594194000	-0.819947000	0.064132000
C	-0.791067000	-0.607110000	0.295982000
O	-1.898853000	0.136576000	-0.202738000
H	0.900358000	1.389169000	1.200835000
H	-0.119528000	2.013371000	-0.109970000
H	1.620546000	1.774556000	-0.368297000
H	0.349014000	-0.057538000	-1.420845000
H	1.513168000	-1.690352000	-0.340992000
H	-0.879955000	-1.665943000	0.029188000
H	-0.708748000	-0.528942000	1.384008000
H	-2.699363000	-0.194551000	0.215300000

Conformer 3-12

Δ*E*=2.52

C	0.709666000	1.386537000	0.101109000
C	0.469055000	-0.038572000	-0.342151000
O	1.609015000	-0.790048000	0.080450000
C	-0.787743000	-0.633146000	0.280271000
O	-1.978427000	-0.002995000	-0.176497000
H	0.808895000	1.424257000	1.186580000
H	-0.111037000	2.032706000	-0.209036000
H	1.627176000	1.767809000	-0.342446000
H	0.367152000	-0.074212000	-1.431554000
H	1.531291000	-1.681809000	-0.276615000
H	-0.876829000	-1.680779000	-0.010166000
H	-0.697308000	-0.591490000	1.369926000
H	-2.039909000	0.858951000	0.246320000

Conformer 3-13

Δ*E*=2.41

C	0.708079000	1.373099000	0.125925000
C	0.467677000	-0.043403000	-0.352413000
O	1.575551000	-0.898621000	-0.068018000
C	-0.788767000	-0.644721000	0.252556000
O	-1.893607000	0.164111000	-0.142965000
H	0.833682000	1.384780000	1.211849000
H	-0.141093000	2.006911000	-0.122136000
H	1.607150000	1.779382000	-0.333895000
H	0.365460000	-0.057025000	-1.437973000
H	1.841144000	-0.736895000	0.844553000
H	-0.893774000	-1.670980000	-0.105239000
H	-0.686144000	-0.663514000	1.344416000
H	-2.703913000	-0.276440000	0.129884000

Conformer 3-14

Δ*E*=2.60

C	0.738047000	1.372908000	0.109402000
C	0.460323000	-0.044094000	-0.346681000
O	1.562792000	-0.914797000	-0.088261000
C	-0.795322000	-0.616986000	0.298869000
O	-1.964468000	0.128143000	-0.027305000
H	0.917364000	1.387216000	1.187547000
H	-0.118451000	2.012685000	-0.095614000
H	1.615811000	1.772387000	-0.395095000
H	0.335659000	-0.066347000	-1.432094000
H	1.847660000	-0.762128000	0.820185000
H	-0.893449000	-1.667518000	0.016909000
H	-0.698320000	-0.564959000	1.385064000
H	-2.211154000	-0.089071000	-0.931921000

Conformer 3-15

Δ*E*=2.60

C	0.751245000	1.379248000	0.107680000
C	0.461976000	-0.036186000	-0.332175000
O	1.590865000	-0.826718000	0.042996000
C	-0.795030000	-0.591764000	0.325246000
O	-1.968877000	0.116699000	-0.053507000
H	0.951093000	1.389838000	1.179195000
H	-0.106112000	2.017097000	-0.097564000
H	1.623833000	1.769071000	-0.412559000
H	0.329167000	-0.063603000	-1.421949000
H	1.458860000	-1.722013000	-0.288656000
H	-0.888461000	-1.659497000	0.096045000
H	-0.707647000	-0.486738000	1.406004000
H	-2.145782000	-0.071785000	-0.980939000

Conformer 3-16

Δ*E*=2.68

C	0.692371000	1.380180000	0.120511000
C	0.468015000	-0.045736000	-0.344636000
O	1.521821000	-0.908446000	0.086741000
C	-0.790706000	-0.656541000	0.248453000
O	-1.969271000	0.016837000	-0.177403000
H	0.753420000	1.412335000	1.209057000
H	-0.114688000	2.030684000	-0.217424000
H	1.622342000	1.777928000	-0.287641000
H	0.376860000	-0.070252000	-1.435370000
H	2.351883000	-0.565625000	-0.260719000
H	-0.879958000	-1.685341000	-0.093895000
H	-0.700885000	-0.666355000	1.338636000
H	-2.047453000	0.832077000	0.326686000

Conformer 3-17

Δ*E*=2.99

C	0.692339000	1.383157000	0.104919000
C	0.470059000	-0.044024000	-0.357266000
O	1.591367000	-0.873207000	-0.050157000
C	-0.786414000	-0.662517000	0.241500000
O	-1.977441000	0.010165000	-0.148503000
H	0.762360000	1.422566000	1.195390000
H	-0.123699000	2.032334000	-0.212925000
H	1.617563000	1.773604000	-0.314507000
H	0.384563000	-0.072014000	-1.443764000
H	1.774637000	-0.783398000	0.892483000
H	-0.880124000	-1.685736000	-0.115725000
H	-0.686422000	-0.692369000	1.333819000
H	-2.016185000	0.849654000	0.319602000

Conformer 3-18

Δ*E*=3.29

C	-1.684660000	-0.764061000	-0.018316000
C	-0.436174000	0.049162000	-0.314680000
O	-0.535147000	1.362470000	0.236962000
C	0.784381000	-0.585410000	0.307811000
O	1.945191000	0.010621000	-0.258254000
H	-1.814677000	-0.870910000	1.058654000
H	-1.622644000	-1.754604000	-0.469177000
H	-2.566792000	-0.264212000	-0.417788000
H	-0.266749000	0.108421000	-1.394780000
H	-1.187371000	1.853069000	-0.273640000
H	0.736584000	-0.421343000	1.388143000
H	0.767844000	-1.661745000	0.111073000
H	2.692174000	-0.171539000	0.318953000

Conformer 3-19

Δ*E*=3.67

C	1.694214000	-0.738376000	0.011415000
C	0.434463000	0.051010000	0.324846000
O	0.496540000	1.368628000	-0.212778000
C	-0.779490000	-0.617361000	-0.287973000
O	-2.011075000	-0.000533000	0.050205000
H	1.819497000	-0.823903000	-1.067799000
H	1.647914000	-1.737936000	0.443896000
H	2.572209000	-0.236172000	0.418387000
H	0.292435000	0.100133000	1.413353000
H	1.260766000	1.811692000	0.171231000
H	-0.700854000	-0.549685000	-1.372753000
H	-0.776679000	-1.675272000	-0.004788000
H	-2.094126000	-0.005262000	1.009330000

Conformer 3-20

Δ*E*=3.67

C	-1.064792000	1.191211000	-0.136896000
C	-0.568400000	-0.110972000	0.453131000
O	-1.013873000	-1.145888000	-0.420682000
C	0.937945000	-0.124599000	0.637705000
O	1.566719000	0.188918000	-0.597059000
H	-0.628781000	1.336709000	-1.122109000
H	-0.785693000	2.028849000	0.501687000
H	-2.148622000	1.163367000	-0.227852000
H	-1.005093000	-0.252387000	1.450079000
H	-0.644522000	-1.981670000	-0.115402000
H	1.195407000	0.607656000	1.410737000
H	1.247142000	-1.114910000	0.993130000
H	2.518872000	0.134311000	-0.471977000

Conformer 3-21

Δ*E*=4.04

C	-0.987887000	1.233739000	-0.094987000
C	-0.558478000	-0.114195000	0.456955000
O	-0.969927000	-1.181344000	-0.393670000
C	0.947796000	-0.228553000	0.616375000
O	1.684557000	0.068036000	-0.560741000
H	-0.597928000	1.366088000	-1.103483000
H	-0.633391000	2.046288000	0.541366000
H	-2.075233000	1.300978000	-0.142045000
H	-0.987732000	-0.250669000	1.457899000
H	-1.907738000	-1.069927000	-0.582577000
H	1.258769000	0.412299000	1.447886000
H	1.190727000	-1.259337000	0.867368000
H	1.626898000	1.014801000	-0.721183000

Conformer 3-22

Δ*E*=3.84

C	-1.705372000	-0.727678000	-0.005242000
C	-0.442712000	0.051248000	-0.334977000
O	-0.555297000	1.413997000	0.063240000
C	0.771310000	-0.615920000	0.278374000
O	1.940849000	0.034097000	-0.196815000
H	-1.838168000	-0.800254000	1.076449000
H	-1.665082000	-1.737909000	-0.412500000
H	-2.570673000	-0.216545000	-0.421423000
H	-0.296606000	0.094151000	-1.414914000
H	-0.624944000	1.435295000	1.024919000
H	0.696842000	-0.548328000	1.371530000
H	0.769441000	-1.675441000	0.002365000
H	2.705418000	-0.381620000	0.213245000

Isomer 4

Conformer 4-1

Δ*E*=0.00

C	-1.914752000	-0.136162000	0.068066000
O	-0.639993000	-0.627087000	-0.327798000
C	0.414343000	0.033214000	0.325121000
C	1.681336000	-0.713644000	-0.014209000
O	0.482702000	1.399126000	-0.033545000
H	-2.655645000	-0.725122000	-0.464221000
H	-2.028606000	0.917591000	-0.185104000
H	-2.056310000	-0.262331000	1.145265000
H	0.228599000	0.050681000	1.403975000
H	1.825394000	-0.709475000	-1.095111000
H	2.532536000	-0.233727000	0.462935000

H	1.611276000	-1.746552000	0.319158000
H	0.715527000	1.432170000	-0.970022000

Conformer 4-2

ΔE=1.65

C	1.655720000	0.150908000	-0.399443000
O	0.828954000	-0.153123000	0.718693000
C	-0.555282000	-0.110480000	0.448427000
C	-1.031285000	1.190118000	-0.157049000
O	-0.941444000	-1.137619000	-0.444163000
H	2.663486000	-0.143978000	-0.121878000
H	1.647009000	1.219530000	-0.620899000
H	1.345633000	-0.406407000	-1.283912000
H	-1.006841000	-0.270042000	1.429551000
H	-0.668194000	1.292603000	-1.177494000
H	-2.118685000	1.197506000	-0.181101000
H	-0.680758000	2.028735000	0.441295000
H	-0.696651000	-1.975289000	-0.033404000

Conformer 4-3

ΔE=1.65

C	-1.902178000	-0.149779000	0.048993000
O	-0.630390000	-0.682103000	-0.271129000
C	0.424886000	0.040565000	0.314343000
C	1.701448000	-0.682266000	-0.019323000
O	0.531637000	1.354716000	-0.200359000
H	-2.643854000	-0.854027000	-0.316099000
H	-2.066247000	0.816030000	-0.434414000
H	-2.017936000	-0.037552000	1.132862000
H	0.251812000	0.091566000	1.398539000
H	1.839092000	-0.668117000	-1.098640000
H	2.543936000	-0.184947000	0.454828000
H	1.644561000	-1.712954000	0.321478000
H	-0.106275000	1.917979000	0.249277000

Conformer 4-4

ΔE=2.43

C	1.915711000	0.028804000	0.103666000
O	0.708261000	-0.509159000	-0.405661000
C	-0.435186000	-0.058013000	0.301812000
C	-0.853506000	1.336764000	-0.095591000
O	-1.488032000	-0.917973000	-0.023881000
H	2.726669000	-0.472251000	-0.416696000
H	2.007512000	-0.157286000	1.178447000
H	1.990603000	1.103731000	-0.076071000
H	-0.204358000	-0.109276000	1.377051000
H	-1.050799000	1.351771000	-1.165567000
H	-1.757659000	1.615984000	0.441804000
H	-0.072785000	2.057205000	0.140254000

H	-1.163127000	-1.818154000	0.097785000

Conformer 4-5

ΔE=3.42

C	1.916517000	-0.000055000	0.091601000
O	0.697731000	-0.538853000	-0.387110000
C	-0.435643000	-0.066005000	0.319341000
C	-0.799935000	1.344754000	-0.100934000
O	-1.481233000	-0.964607000	0.087925000
H	2.715768000	-0.525483000	-0.422918000
H	2.017178000	-0.161012000	1.169377000
H	2.005170000	1.068979000	-0.116745000
H	-0.237447000	-0.118573000	1.395227000
H	-0.996234000	1.360897000	-1.173498000
H	-1.692069000	1.667316000	0.432023000
H	0.005839000	2.044570000	0.114570000
H	-1.635819000	-0.981179000	-0.864607000

Conformer 4-6

ΔE=4.21

C	1.661662000	0.123381000	-0.413279000
O	0.840304000	0.011509000	0.736302000
C	-0.541187000	-0.103855000	0.469937000
C	-1.115784000	1.117664000	-0.223450000
O	-0.851562000	-1.287928000	-0.235574000
H	2.687440000	0.096488000	-0.057762000
H	1.499312000	1.064323000	-0.944216000
H	1.507897000	-0.714279000	-1.096745000
H	-0.979754000	-0.228053000	1.457777000
H	-0.755990000	1.193994000	-1.250470000
H	-2.201193000	1.043503000	-0.246879000
H	-0.831074000	2.021651000	0.311216000
H	-0.864715000	-1.089409000	-1.177991000

Isomer 5

Conformer 5-1

ΔE=0.00

O	-1.495523000	-0.919026000	-0.132969000
C	-1.249040000	0.409619000	0.303415000
C	-0.024947000	1.033284000	-0.352723000
C	1.278823000	0.474425000	0.176424000
O	1.298716000	-0.936996000	-0.086141000
H	-0.643496000	-1.374412000	-0.098532000
H	-2.140392000	0.980764000	0.047021000
H	-1.134796000	0.445860000	1.394375000
H	-0.081013000	0.881086000	-1.431985000
H	-0.026635000	2.110248000	-0.166219000
H	2.129078000	0.957913000	-0.310466000

H	1.346988000	0.654118000	1.253658000
H	2.095702000	-1.311378000	0.302331000

Conformer 5-2

Δ*E*=0.31

O	1.389508000	-0.937950000	0.031928000
C	1.250248000	0.477187000	0.233201000
C	-0.031448000	1.033447000	-0.363177000
C	-1.282552000	0.413938000	0.241438000
O	-1.443414000	-0.955439000	-0.102788000
H	1.551939000	-1.085064000	-0.906622000
H	1.245317000	0.606865000	1.315366000
H	2.123708000	1.000102000	-0.163127000
H	-0.046994000	2.113054000	-0.187440000
H	-0.039642000	0.879208000	-1.445414000
H	-1.256750000	0.538384000	1.331064000
H	-2.170916000	0.924651000	-0.127566000
H	-0.592900000	-1.377521000	0.081848000

Conformer 5-3

Δ*E*=0.82

O	-1.719767000	-0.574079000	0.465299000
C	-1.104192000	0.152182000	-0.602894000
C	-0.000000000	0.993587000	0.000000000
C	1.104192000	0.152181000	0.602894000
O	1.719768000	-0.574078000	-0.465300000
H	-2.339624000	-1.202166000	0.082477000
H	-0.683467000	-0.531764000	-1.342343000
H	-1.836115000	0.797804000	-1.095403000
H	0.423606000	1.634903000	-0.775198000
H	-0.423606000	1.634902000	0.775199000
H	1.836114000	0.797802000	1.095405000
H	0.683467000	-0.531767000	1.342342000
H	2.339622000	-1.202168000	-0.082479000

Conformer 5-4

Δ*E*=1.21

O	1.542781000	-0.768544000	-0.442349000
C	1.169493000	0.235787000	0.498431000
C	0.023482000	1.105783000	-0.003324000
C	-1.140372000	0.277989000	-0.509224000
O	-1.430454000	-0.719363000	0.478617000
H	0.877182000	-1.462196000	-0.375789000
H	0.895504000	-0.220360000	1.450465000
H	2.056392000	0.846631000	0.658314000
H	-0.305969000	1.752729000	0.812461000
H	0.367463000	1.742476000	-0.820328000
H	-2.010680000	0.914913000	-0.682041000
H	-0.869712000	-0.198132000	-1.454601000

H	-2.224422000	-1.190154000	0.206081000

Conformer 5-5

ΔE=1.48

O	-1.492772000	-0.809029000	0.366775000
C	-1.135995000	0.241725000	-0.536117000
C	0.009061000	1.097510000	-0.018731000
C	1.163425000	0.254226000	0.508684000
O	1.569003000	-0.749882000	-0.415525000
H	-1.840981000	-0.408473000	1.170369000
H	-0.833154000	-0.264244000	-1.450349000
H	-2.003923000	0.861914000	-0.771992000
H	0.354279000	1.734419000	-0.835988000
H	-0.339042000	1.755646000	0.783208000
H	2.036686000	0.881999000	0.678008000
H	0.888436000	-0.199857000	1.462771000
H	0.908903000	-1.450880000	-0.369040000

Conformer 5-6

ΔE=1.37

O	1.765425000	-0.539326000	-0.433118000
C	1.095695000	0.163920000	0.615895000
C	-0.008245000	0.976467000	-0.026217000
C	-1.095179000	0.099750000	-0.625379000
O	-1.765210000	-0.684522000	0.363401000
H	2.346670000	-1.193945000	-0.034586000
H	0.665728000	-0.535408000	1.336341000
H	1.793656000	0.823634000	1.138273000
H	-0.444113000	1.644264000	0.722175000
H	0.423907000	1.601981000	-0.810889000
H	-1.811069000	0.710812000	-1.181115000
H	-0.652046000	-0.616064000	-1.313756000
H	-2.278079000	-0.085310000	0.915502000

Conformer 5-7

ΔE=1.72

O	1.850723000	-0.728822000	-0.250218000
C	1.379506000	0.523571000	0.253033000
C	-0.040495000	0.712059000	-0.231614000
C	-0.967316000	-0.368135000	0.282204000
O	-2.287232000	-0.035390000	-0.151994000
H	2.763465000	-0.843357000	0.031942000
H	1.406329000	0.527996000	1.347743000
H	2.006824000	1.342791000	-0.107032000
H	-0.407244000	1.682238000	0.108873000
H	-0.057685000	0.713282000	-1.321958000
H	-0.660428000	-1.339796000	-0.107325000
H	-0.920739000	-0.404687000	1.376149000
H	-2.868626000	-0.769740000	0.067565000

Conformer 5-8
ΔE=1.84

O	1.850011000	-0.734364000	-0.244397000
C	1.382974000	0.530979000	0.228821000
C	-0.041660000	0.702725000	-0.251991000
C	-0.980407000	-0.329511000	0.349127000
O	-2.334226000	-0.127620000	-0.054920000
H	2.755402000	-0.855376000	0.058075000
H	1.415082000	0.565922000	1.322855000
H	2.008869000	1.339752000	-0.156749000
H	-0.394394000	1.700563000	0.014274000
H	-0.055268000	0.624683000	-1.341884000
H	-0.643216000	-1.336992000	0.100634000
H	-0.985684000	-0.235831000	1.435261000
H	-2.392511000	-0.332006000	-0.993673000

Conformer 5-9
ΔE=1.90

O	1.942566000	-0.747212000	-0.116748000
C	1.387174000	0.513901000	0.257340000
C	-0.034532000	0.701840000	-0.240934000
C	-0.984040000	-0.325166000	0.334891000
O	-2.278911000	-0.060803000	-0.207025000
H	2.016490000	-0.760452000	-1.076831000
H	1.411702000	0.523181000	1.347025000
H	2.020026000	1.332546000	-0.094746000
H	-0.382367000	1.701221000	0.029803000
H	-0.059274000	0.638148000	-1.331807000
H	-0.649094000	-1.331076000	0.075330000
H	-0.997825000	-0.240066000	1.426517000
H	-2.880516000	-0.742840000	0.107105000

Conformer 5-10
ΔE=2.09

O	-1.802732000	-0.657145000	0.332192000
C	-1.089986000	0.113427000	-0.635192000
C	0.000000000	0.960924000	0.000003000
C	1.089986000	0.113422000	0.635193000
O	1.802732000	-0.657143000	-0.332196000
H	-2.319427000	-0.048794000	0.870581000
H	-0.643913000	-0.611880000	-1.312667000
H	-1.776265000	0.741195000	-1.209550000
H	0.439933000	1.613322000	-0.760874000
H	-0.439932000	1.613317000	0.760886000
H	1.776265000	0.741186000	1.209556000
H	0.643913000	-0.611889000	1.312662000
H	2.319424000	-0.048787000	-0.870584000

Conformer 5-11
Δ*E*=2.09

O	1.855714000	-0.719930000	-0.233961000
C	1.375858000	0.539807000	0.237576000
C	-0.046184000	0.700075000	-0.255965000
C	-0.969988000	-0.367389000	0.305132000
O	-2.309219000	-0.230167000	-0.164192000
H	2.763141000	-0.830339000	0.066425000
H	1.398586000	0.572212000	1.332132000
H	1.997504000	1.355359000	-0.140796000
H	-0.405660000	1.689526000	0.041418000
H	-0.061611000	0.653984000	-1.345466000
H	-0.643324000	-1.351392000	-0.020268000
H	-0.938898000	-0.347876000	1.400213000
H	-2.639810000	0.624345000	0.131112000

Conformer 5-12
Δ*E*=2.28

O	1.946203000	-0.734732000	-0.092875000
C	1.383850000	0.533458000	0.238963000
C	-0.040636000	0.690955000	-0.266410000
C	-0.982997000	-0.331296000	0.344443000
O	-2.302736000	-0.247045000	-0.188307000
H	2.017392000	-0.782797000	-1.052087000
H	1.405703000	0.578380000	1.327944000
H	2.011277000	1.345234000	-0.138449000
H	-0.386489000	1.701683000	-0.027881000
H	-0.063942000	0.592320000	-1.354714000
H	-0.638504000	-1.337607000	0.119898000
H	-0.995264000	-0.219479000	1.433796000
H	-2.659208000	0.617782000	0.038978000

Conformer 5-13
Δ*E*=2.38

O	1.944057000	-0.747488000	-0.091323000
C	1.385571000	0.526322000	0.232153000
C	-0.037623000	0.688245000	-0.274748000
C	-0.993074000	-0.299458000	0.372176000
O	-2.337447000	-0.130925000	-0.075495000
H	2.080417000	-0.774752000	-1.043958000
H	1.404032000	0.578319000	1.320874000
H	2.018623000	1.332168000	-0.147767000
H	-0.382719000	1.704061000	-0.070132000
H	-0.051558000	0.557232000	-1.361838000
H	-0.649038000	-1.322188000	0.207406000
H	-1.022941000	-0.128543000	1.448188000
H	-2.378944000	-0.409637000	-0.995709000

Conformer 5-14

Δ*E*=2.71

O	-2.339692000	-0.127785000	-0.067142000
C	-0.988371000	-0.338766000	0.330971000
C	-0.041641000	0.710938000	-0.222206000
C	1.389946000	0.516837000	0.250010000
O	1.996590000	-0.661617000	-0.280937000
H	-2.375624000	-0.189907000	-1.027414000
H	-0.998754000	-0.293780000	1.420491000
H	-0.661495000	-1.342184000	0.039606000
H	-0.055347000	0.682418000	-1.314646000
H	-0.392887000	1.696531000	0.090790000
H	2.009969000	1.341067000	-0.096866000
H	1.430131000	0.510952000	1.344130000
H	1.629223000	-1.423935000	0.175884000

Conformer 5-15

Δ*E*=2.66

O	-2.451123000	-0.270266000	-0.088953000
C	-1.256047000	0.496464000	0.025257000
C	-0.004052000	-0.360210000	-0.035669000
C	1.252815000	0.480786000	0.018604000
O	2.367673000	-0.408565000	-0.007569000
H	-2.460988000	-0.907354000	0.632906000
H	-1.281236000	1.191429000	-0.813655000
H	-1.265547000	1.088622000	0.946383000
H	0.009061000	-1.059889000	0.803757000
H	-0.009170000	-0.946806000	-0.954492000
H	1.282455000	1.162081000	-0.837543000
H	1.263163000	1.083357000	0.932566000
H	3.173573000	0.116976000	0.013109000

Conformer 5-16

Δ*E*=2.78

O	2.448945000	0.285355000	-0.085503000
C	1.257570000	-0.491455000	-0.004328000
C	-0.000000000	0.360516000	0.000002000
C	-1.257570000	-0.491456000	0.004324000
O	-2.448946000	0.285353000	0.085504000
H	2.484587000	0.851261000	0.692631000
H	1.275279000	-1.135226000	-0.883273000
H	1.276991000	-1.137620000	0.879290000
H	-0.010819000	1.005922000	0.881599000
H	0.010818000	1.005929000	-0.881590000
H	-1.276990000	-1.137614000	-0.879300000
H	-1.275280000	-1.135233000	0.883263000
H	-2.484574000	0.851286000	-0.692612000

Conformer 5-17

Δ*E*=2.74

O	1.856560000	-0.577449000	-0.382187000
C	1.123257000	0.155455000	0.596709000
C	0.000000000	0.987148000	-0.000001000
C	-1.123257000	0.155453000	-0.596709000
O	-1.856560000	-0.577448000	0.382187000
H	1.257333000	-1.193172000	-0.815619000
H	0.740946000	-0.515756000	1.374541000
H	1.853770000	0.806050000	1.073001000
H	-0.420769000	1.628302000	0.777542000
H	0.420769000	1.628301000	-0.777544000
H	-1.853769000	0.806048000	-1.073004000
H	-0.740945000	-0.515759000	-1.374540000
H	-1.257334000	-1.193168000	0.815624000

Conformer 5-18

Δ*E*=2.67

O	2.006987000	-0.656252000	-0.278746000
C	1.387625000	0.507920000	0.269164000
C	-0.037231000	0.711787000	-0.215109000
C	-0.982730000	-0.357160000	0.286653000
O	-2.289287000	-0.032690000	-0.183018000
H	1.609580000	-1.431136000	0.129125000
H	1.417068000	0.478478000	1.363462000
H	2.008821000	1.340828000	-0.053624000
H	-0.396549000	1.681889000	0.135460000
H	-0.053053000	0.723709000	-1.305323000
H	-0.679532000	-1.339626000	-0.088955000
H	-0.962262000	-0.385226000	1.382064000
H	-2.891663000	-0.732659000	0.087653000

Conformer 5-19

Δ*E*=2.65

O	-2.370780000	-0.398813000	-0.000009000
C	-1.251096000	0.484839000	0.000010000
C	0.000000000	-0.365622000	-0.000001000
C	1.251096000	0.484839000	-0.000008000
O	2.370780000	-0.398813000	0.000011000
H	-3.173484000	0.131835000	0.000010000
H	-1.269239000	1.127638000	-0.886051000
H	-1.269244000	1.127608000	0.886093000
H	0.000008000	-1.008742000	0.880246000
H	-0.000008000	-1.008739000	-0.880250000
H	1.269246000	1.127609000	-0.886090000
H	1.269237000	1.127637000	0.886054000
H	3.173484000	0.131835000	-0.000037000

Conformer 5-20
Δ*E*=2.88

O	2.008714000	-0.647800000	-0.261951000
C	1.383230000	0.525745000	0.254252000
C	-0.043952000	0.702035000	-0.238935000
C	-0.981969000	-0.363285000	0.299456000
O	-2.315783000	-0.221740000	-0.178040000
H	1.624442000	-1.414457000	0.173416000
H	1.409118000	0.525868000	1.349106000
H	1.999286000	1.354459000	-0.089029000
H	-0.399301000	1.686915000	0.080888000
H	-0.057822000	0.679484000	-1.329084000
H	-0.669697000	-1.351210000	-0.037766000
H	-0.960272000	-0.359207000	1.395564000
H	-2.633060000	0.647501000	0.088200000

Conformer 5-21
Δ*E*=2.99

O	-2.450920000	-0.274246000	-0.103723000
C	-1.257046000	0.488292000	0.046184000
C	-0.000002000	-0.358854000	-0.054352000
C	1.257043000	0.488292000	0.046161000
O	2.450915000	-0.274262000	-0.103674000
H	-2.481992000	-0.918023000	0.611400000
H	-1.280578000	1.218908000	-0.761784000
H	-1.268307000	1.040437000	0.991658000
H	0.000008000	-1.102342000	0.749962000
H	-0.000010000	-0.898769000	-1.001354000
H	1.280597000	1.218859000	-0.761851000
H	1.268286000	1.040496000	0.991601000
H	2.482056000	-0.917883000	0.611586000

Conformer 5-22
Δ*E*=4.98

O	1.386753000	-0.986391000	-0.038109000
C	1.355206000	0.427164000	0.132309000
C	0.026858000	0.957094000	-0.365048000
C	-1.184391000	0.422958000	0.382155000
O	-1.612874000	-0.779682000	-0.251862000
H	2.239140000	-1.303824000	0.276541000
H	1.475282000	0.687943000	1.190083000
H	2.167672000	0.902448000	-0.426021000
H	0.054216000	2.044911000	-0.268993000
H	-0.094657000	0.722648000	-1.423745000
H	-0.923659000	0.238767000	1.429772000
H	-1.988726000	1.164122000	0.357815000
H	-2.306344000	-1.171735000	0.287812000

Isomer 6

Conformer 6-1

Δ*E*=0.00

C	-2.277275000	-0.114985000	-0.181791000
C	-0.894617000	-0.278348000	0.401923000
O	-0.015269000	0.599461000	-0.302319000
C	1.296638000	0.546141000	0.180685000
O	1.939995000	-0.679148000	-0.088256000
H	-2.613137000	0.915489000	-0.083180000
H	-2.982140000	-0.763752000	0.336946000
H	-2.275357000	-0.378895000	-1.237673000
H	-0.882801000	-0.013842000	1.465365000
H	-0.544382000	-1.307782000	0.305384000
H	1.815067000	1.381075000	-0.289995000
H	1.316654000	0.644713000	1.268551000
H	2.019814000	-0.756360000	-1.045697000

Conformer 6-2

Δ*E*=1.09

C	2.030032000	0.277109000	-0.374851000
C	0.935624000	-0.655175000	0.107352000
O	-0.060277000	0.046223000	0.861346000
C	-1.043999000	0.630851000	0.054890000
O	-1.904080000	-0.317226000	-0.536259000
H	1.639291000	1.033731000	-1.053830000
H	2.793342000	-0.288616000	-0.909707000
H	2.496424000	0.779303000	0.470653000
H	0.460733000	-1.178436000	-0.725032000
H	1.343060000	-1.402198000	0.786111000
H	-1.587982000	1.311406000	0.709251000
H	-0.609367000	1.177592000	-0.783519000
H	-2.350594000	-0.781458000	0.181042000

Conformer 6-3

Δ*E*=1.37

C	1.759645000	0.698242000	-0.105369000
C	1.047911000	-0.567452000	0.330111000
O	-0.099229000	-0.849606000	-0.477654000
C	-1.307599000	-0.426999000	0.081396000
O	-1.443343000	0.975920000	0.150532000
H	2.016992000	0.635461000	-1.161572000
H	2.680801000	0.821344000	0.465590000
H	1.129620000	1.569395000	0.054884000
H	1.707513000	-1.428472000	0.223143000
H	0.743432000	-0.499023000	1.378791000
H	-2.084408000	-0.871463000	-0.540580000
H	-1.400989000	-0.767829000	1.114664000

H	-1.452122000	1.307329000	-0.754763000

Conformer 6-4

ΔE=1.83

C	2.293688000	0.067215000	-0.134768000
C	0.875793000	0.378057000	0.282501000
O	0.029830000	-0.637101000	-0.241077000
C	-1.287466000	-0.541299000	0.222005000
O	-1.991786000	0.570085000	-0.284666000
H	2.600505000	-0.900130000	0.257739000
H	2.973500000	0.830948000	0.241167000
H	2.365920000	0.040026000	-1.220144000
H	0.784781000	0.398074000	1.376419000
H	0.571904000	1.355551000	-0.104350000
H	-1.798391000	-1.424062000	-0.148853000
H	-1.292142000	-0.523234000	1.318899000
H	-1.802526000	1.335124000	0.266638000

Conformer 6-5

ΔE=2.30

C	-2.336464000	-0.373753000	-0.014743000
C	-1.119648000	0.518601000	0.047269000
O	0.035705000	-0.301791000	-0.057415000
C	1.215646000	0.468425000	-0.000649000
O	2.318381000	-0.376262000	-0.074021000
H	-2.354802000	-0.920789000	-0.955084000
H	-3.244842000	0.222650000	0.061241000
H	-2.320445000	-1.091007000	0.803500000
H	-1.130376000	1.247245000	-0.771941000
H	-1.093811000	1.077970000	0.990316000
H	1.280355000	1.138658000	-0.861789000
H	1.211778000	1.058945000	0.926171000
H	2.262251000	-0.988894000	0.667815000

Conformer 6-6

ΔE=3.44

C	-1.788691000	0.661182000	-0.166615000
C	-1.023450000	-0.522565000	0.393744000
O	0.083889000	-0.875817000	-0.432365000
C	1.318509000	-0.422302000	0.041057000
O	1.491102000	0.976882000	-0.035681000
H	-1.160968000	1.547929000	-0.215617000
H	-2.659586000	0.873841000	0.454616000
H	-2.129846000	0.433646000	-1.174737000
H	-0.673163000	-0.318556000	1.412880000
H	-1.668906000	-1.399966000	0.439960000
H	1.466557000	-0.758766000	1.073913000
H	2.064519000	-0.860283000	-0.614575000
H	1.123258000	1.375736000	0.758816000

Conformer 6-7
Δ*E*=3.46

C	-2.031883000	0.565900000	-0.124068000
C	-1.176240000	-0.639454000	0.222933000
O	0.124947000	-0.585482000	-0.351314000
C	0.940295000	0.406823000	0.232610000
O	2.264871000	0.167394000	-0.123410000
H	-1.640867000	1.479783000	0.320709000
H	-3.045416000	0.420635000	0.249960000
H	-2.075347000	0.696019000	-1.204230000
H	-1.092110000	-0.748323000	1.311071000
H	-1.623505000	-1.548790000	-0.173674000
H	0.702958000	1.400322000	-0.150827000
H	0.794404000	0.384824000	1.322559000
H	2.468304000	-0.739375000	0.133373000

Conformer 6-8
Δ*E*=3.56

C	1.840691000	-0.544463000	0.328577000
C	0.974177000	0.262981000	-0.623029000
O	-0.072430000	0.979245000	0.027087000
C	-1.070980000	0.168872000	0.583791000
O	-1.803607000	-0.563298000	-0.374192000
H	2.220115000	0.091920000	1.126329000
H	2.688138000	-0.959842000	-0.216827000
H	1.302257000	-1.377543000	0.778598000
H	1.570925000	1.031144000	-1.110230000
H	0.556813000	-0.372785000	-1.409159000
H	-0.650852000	-0.509078000	1.332657000
H	-1.767671000	0.857077000	1.051792000
H	-1.374754000	-1.412801000	-0.512345000

Conformer 6-9
Δ*E*=3.67

C	-1.543064000	0.885651000	0.023344000
C	-1.209828000	-0.595810000	0.082285000
O	0.078679000	-0.919678000	-0.429058000
C	1.146978000	-0.412977000	0.326374000
O	1.665786000	0.799395000	-0.167050000
H	-0.942611000	1.462571000	0.728447000
H	-2.587499000	1.039856000	0.295437000
H	-1.385707000	1.271754000	-0.982659000
H	-1.297776000	-0.961688000	1.111187000
H	-1.905868000	-1.160833000	-0.534597000
H	0.837210000	-0.310327000	1.373575000
H	1.963913000	-1.123915000	0.243792000
H	0.998104000	1.483672000	-0.058339000

Conformer 6-10
ΔE=3.92

C	-2.024699000	0.593455000	-0.149056000
C	-1.195224000	-0.627958000	0.210143000
O	0.122419000	-0.600407000	-0.327024000
C	0.955869000	0.341783000	0.310608000
O	2.278493000	0.095970000	-0.051551000
H	-1.641790000	1.499208000	0.317856000
H	-3.051053000	0.453107000	0.190535000
H	-2.034670000	0.735481000	-1.228703000
H	-1.141471000	-0.748730000	1.298565000
H	-1.650332000	-1.525548000	-0.204015000
H	0.653276000	1.358228000	0.035370000
H	0.902128000	0.221081000	1.396372000
H	2.340936000	0.198994000	-1.007558000

Isomer 7

Conformer 7-1
1 0.00

C	2.167051000	-0.220676000	0.082374000
O	0.826152000	-0.275264000	-0.366560000
C	0.034552000	0.737806000	0.231586000
C	-1.386643000	0.503654000	-0.217188000
O	-1.858279000	-0.764985000	0.209857000
H	2.707308000	-1.022804000	-0.412491000
H	2.222649000	-0.358806000	1.166211000
H	2.626915000	0.738095000	-0.175158000
H	0.100725000	0.667489000	1.323545000
H	0.380914000	1.730413000	-0.081717000
H	-2.043110000	1.250861000	0.225925000
H	-1.439974000	0.593957000	-1.306097000
H	-1.188165000	-1.401914000	-0.067218000

Conformer 7-2
ΔE=1.47

C	-1.809447000	-0.437304000	0.362550000
O	-0.933968000	-0.002110000	-0.665288000
C	0.017306000	0.951734000	-0.206474000
C	1.138016000	0.280118000	0.561483000
O	1.738052000	-0.740249000	-0.220871000
H	-2.535785000	-1.098375000	-0.101709000
H	-2.329814000	0.413458000	0.811845000
H	-1.282978000	-0.984427000	1.148272000
H	0.419818000	1.419335000	-1.103874000
H	-0.471238000	1.715516000	0.409697000
H	1.911861000	1.011939000	0.792085000

H	0.769594000	-0.126285000	1.508628000
H	1.010621000	-1.179572000	-0.681020000

Conformer 7-3

ΔE=2.35

C	2.330358000	0.382405000	0.000000000
O	1.175631000	-0.433428000	0.000000000
C	0.000000000	0.350177000	0.000000000
C	-1.176987000	-0.596386000	0.000000000
O	-2.347982000	0.216207000	0.000000000
H	3.193170000	-0.277739000	0.000000000
H	2.361060000	1.019489000	0.889373000
H	2.361060000	1.019489000	-0.889373000
H	-0.040283000	0.992295000	0.887412000
H	-0.040283000	0.992295000	-0.887412000
H	-1.130157000	-1.231304000	-0.887513000
H	-1.130157000	-1.231304000	0.887513000
H	-3.115823000	-0.362628000	0.000000000

Conformer 7-4

ΔE=2.55

C	-2.353976000	-0.208252000	0.008062000
O	-1.141134000	0.516772000	-0.049363000
C	-0.025260000	-0.344247000	0.034579000
C	1.224293000	0.511944000	-0.004028000
O	2.399338000	-0.286231000	0.091615000
H	-3.164928000	0.511313000	-0.060747000
H	-2.437188000	-0.760078000	0.949367000
H	-2.427255000	-0.917278000	-0.822631000
H	-0.047967000	-0.925495000	0.963535000
H	-0.024614000	-1.052218000	-0.806233000
H	1.222732000	1.123582000	-0.908821000
H	1.236068000	1.178548000	0.855240000
H	2.507179000	-0.759374000	-0.739408000

Conformer 7-5

ΔE=2.62

C	2.213010000	-0.241410000	0.092924000
O	0.903089000	-0.244752000	-0.436126000
C	0.064361000	0.636534000	0.280311000
C	-1.342919000	0.507166000	-0.236853000
O	-1.882982000	-0.719150000	0.243936000
H	2.805647000	-0.927021000	-0.506254000
H	2.216669000	-0.575503000	1.135207000
H	2.657626000	0.758445000	0.043500000
H	0.078839000	0.395066000	1.350236000
H	0.407663000	1.671918000	0.154935000
H	-1.930676000	1.357339000	0.122871000
H	-1.319720000	0.529952000	-1.328953000

H	-2.683612000	-0.912730000	-0.252311000

Conformer 7-6

ΔE=3.06

C	-1.655458000	0.737824000	0.057485000
O	-1.146465000	-0.515227000	-0.378828000
C	-0.012183000	-0.946257000	0.347434000
C	1.295787000	-0.422846000	-0.198928000
O	1.408773000	0.965029000	0.116441000
H	-2.569730000	0.911970000	-0.503787000
H	-0.947693000	1.545685000	-0.123621000
H	-1.891686000	0.704774000	1.125531000
H	-0.102252000	-0.668942000	1.404223000
H	0.002426000	-2.035081000	0.275743000
H	2.114508000	-0.990138000	0.256577000
H	1.314316000	-0.580717000	-1.280249000
H	2.212772000	1.301708000	-0.291271000

Conformer 7-7

ΔE=3.01

C	2.219428000	-0.222712000	0.084725000
O	0.895075000	-0.273653000	-0.405429000
C	0.062803000	0.632583000	0.280985000
C	-1.350572000	0.511626000	-0.237151000
O	-1.962617000	-0.734094000	0.063017000
H	2.803829000	-0.936448000	-0.488938000
H	2.257677000	-0.493315000	1.144978000
H	2.648203000	0.777577000	-0.037016000
H	0.087044000	0.427009000	1.361749000
H	0.413974000	1.663520000	0.132869000
H	-1.938933000	1.346813000	0.157089000
H	-1.340176000	0.581926000	-1.323174000
H	-1.981231000	-0.834089000	1.020390000

Conformer 7-8

ΔE=3.60

C	1.592265000	-0.785873000	0.023569000
O	1.204373000	0.544827000	-0.286659000
C	0.016780000	0.956650000	0.356584000
C	-1.258164000	0.417743000	-0.265040000
O	-1.493133000	-0.965845000	-0.009745000
H	2.587775000	-0.921753000	-0.390689000
H	0.908778000	-1.518383000	-0.403958000
H	1.633057000	-0.930725000	1.108371000
H	0.050613000	0.691314000	1.423393000
H	0.000363000	2.045468000	0.280407000
H	-2.101931000	1.024106000	0.081602000
H	-1.192187000	0.512074000	-1.347707000
H	-1.681671000	-1.065071000	0.929137000

Conformer 7-9

Δ*E*=3.88

C	-2.033953000	-0.510701000	0.182026000
O	-1.267403000	0.551928000	-0.357118000
C	0.018495000	0.643877000	0.228529000
C	0.996611000	-0.365727000	-0.355408000
O	2.286199000	-0.227660000	0.233320000
H	-3.019696000	-0.450259000	-0.270751000
H	-2.128468000	-0.408942000	1.267382000
H	-1.601189000	-1.488180000	-0.043935000
H	0.376749000	1.654311000	0.023376000
H	-0.035775000	0.515466000	1.315307000
H	0.681804000	-1.383284000	-0.133830000
H	1.032148000	-0.250054000	-1.441366000
H	2.657136000	0.612107000	-0.056680000

Conformer 7-10

Δ*E*=3.83

C	-2.030905000	-0.523778000	0.208277000
O	-1.270108000	0.514970000	-0.380863000
C	0.009667000	0.652778000	0.210410000
C	1.012540000	-0.313971000	-0.391491000
O	2.243946000	-0.087811000	0.290212000
H	-2.996848000	-0.530431000	-0.289228000
H	-2.176523000	-0.339948000	1.276960000
H	-1.561490000	-1.502673000	0.081026000
H	0.340477000	1.673272000	0.023522000
H	-0.040481000	0.500657000	1.293926000
H	0.675832000	-1.345174000	-0.258056000
H	1.103211000	-0.113598000	-1.461434000
H	2.917317000	-0.649550000	-0.104684000

Conformer 7-11

Δ*E*=4.11

C	-2.024656000	-0.539343000	0.171746000
O	-1.275494000	0.554064000	-0.326535000
C	0.014583000	0.640635000	0.251268000
C	1.012275000	-0.289088000	-0.424146000
O	2.313411000	-0.137117000	0.135510000
H	-3.004888000	-0.490204000	-0.294247000
H	-2.138758000	-0.468313000	1.257988000
H	-1.566230000	-1.500825000	-0.072518000
H	0.350256000	1.669391000	0.129494000
H	-0.032736000	0.422592000	1.326810000
H	0.678601000	-1.328000000	-0.367633000
H	1.098920000	-0.020204000	-1.474577000
H	2.298285000	-0.493230000	1.029675000

Conformer 7-12
Δ*E*=4.15

C	-1.768416000	-0.599116000	0.266695000
O	-1.046780000	0.200536000	-0.647695000
C	-0.031877000	0.965443000	-0.031348000
C	1.088339000	0.157192000	0.586543000
O	1.717214000	-0.594558000	-0.443105000
H	-2.630419000	-0.988895000	-0.267812000
H	-2.113739000	-0.007375000	1.122002000
H	-1.174159000	-1.439495000	0.633717000
H	0.379097000	1.596558000	-0.818024000
H	-0.461266000	1.607691000	0.750094000
H	1.786527000	0.862974000	1.049069000
H	0.704591000	-0.498275000	1.373202000
H	2.417617000	-1.122117000	-0.047188000

Isomer 8

Conformer 8-1
Δ*E*=0.00

C	-0.000000000	1.774796000	-0.579268000
O	0.790469000	0.865690000	0.175619000
C	0.000000000	-0.000000000	0.943046000
O	-0.790469000	-0.865690000	0.175619000
C	0.000000000	-1.774796000	-0.579268000
H	0.688823000	2.446175000	-1.083073000
H	-0.655767000	2.351677000	0.079329000
H	-0.611013000	1.252067000	-1.315851000
H	0.704447000	-0.562951000	1.561115000
H	-0.704447000	0.562951000	1.561115000
H	0.611013000	-1.252067000	-1.315851000
H	0.655767000	-2.351677000	0.079329000
H	-0.688823000	-2.446175000	-1.083073000

Conformer 8-2
Δ*E*=2.59

C	-2.155789000	-0.243515000	0.109284000
O	-0.836781000	-0.302213000	-0.399575000
C	-0.028060000	0.685421000	0.204137000
O	1.265659000	0.587750000	-0.271951000
C	1.901197000	-0.617571000	0.137460000
H	-2.726305000	-1.021229000	-0.389125000
H	-2.616814000	0.727822000	-0.095692000
H	-2.172876000	-0.419307000	1.189661000
H	-0.389130000	1.684077000	-0.058016000
H	-0.057873000	0.549048000	1.298322000
H	1.864626000	-0.717066000	1.226484000

H	1.427740000	-1.485226000	-0.319698000
H	2.935522000	-0.548421000	-0.185014000

Conformer 8-3

Δ*E*=3.64

C	1.534235000	0.875887000	0.001793000
O	1.316774000	-0.519930000	-0.120839000
C	0.040025000	-0.901159000	0.295258000
O	-0.978644000	-0.364390000	-0.516428000
C	-1.798075000	0.594553000	0.134806000
H	2.575638000	1.056321000	-0.247294000
H	1.344567000	1.209473000	1.027757000
H	0.896843000	1.435596000	-0.684235000
H	0.017709000	-1.984678000	0.201984000
H	-0.126539000	-0.596790000	1.338654000
H	-2.349317000	0.146759000	0.965180000
H	-1.214360000	1.438072000	0.511061000
H	-2.506689000	0.954115000	-0.606109000

Conformer 8-4

Δ*E*=5.38

C	0.000000000	2.306566000	0.218553000
O	0.000000000	1.113541000	-0.543376000
C	0.000000000	0.000000000	0.293846000
O	-0.000000000	-1.113541000	-0.543376000
C	-0.000000000	-2.306566000	0.218553000
H	0.000000000	3.131694000	-0.486519000
H	0.890112000	2.372409000	0.851849000
H	-0.890112000	2.372409000	0.851849000
H	0.897346000	-0.000000000	0.936974000
H	-0.897346000	0.000000000	0.936974000
H	0.890112000	-2.372409000	0.851849000
H	-0.890112000	-2.372409000	0.851849000
H	-0.000000000	-3.131694000	-0.486519000

Isomer 9

Conformer 9-1

Δ*E*=0.00

C	-0.496589000	1.453406000	-0.100778000
C	-0.397706000	0.007425000	0.340067000
C	-1.634983000	-0.795293000	-0.009408000
O	0.656016000	-0.673913000	-0.349043000
O	1.900840000	-0.029359000	0.055497000
H	-0.708554000	1.500339000	-1.168831000
H	0.433816000	1.980132000	0.097185000
H	-1.301058000	1.953018000	0.439424000
H	-0.195639000	-0.049698000	1.414207000
H	-1.799543000	-0.776916000	-1.086256000

H	-1.534217000	-1.829561000	0.313869000
H	-2.503069000	-0.357045000	0.481100000
H	2.329078000	-0.787322000	0.478389000

Conformer 9-2

ΔE=0.18

C	-1.654479000	-0.758266000	0.028566000
C	-0.402653000	0.012580000	-0.339593000
C	-0.457242000	1.459797000	0.106398000
O	0.645287000	-0.707114000	0.319103000
O	1.900937000	-0.173406000	-0.193531000
H	-1.803245000	-0.738606000	1.107978000
H	-1.581026000	-1.793549000	-0.297668000
H	-2.519520000	-0.298963000	-0.447763000
H	-0.221070000	-0.047277000	-1.416313000
H	-0.632790000	1.511731000	1.181419000
H	0.472161000	1.973160000	-0.130008000
H	-1.270361000	1.976778000	-0.403360000
H	2.272302000	0.176214000	0.628912000

Conformer 9-3

ΔE=1.08

C	-0.887245000	1.302605000	-0.240220000
C	-0.521515000	0.013242000	0.469860000
C	-1.004093000	-1.223064000	-0.263506000
O	0.882699000	-0.062996000	0.753099000
O	1.574413000	-0.131350000	-0.530142000
H	-0.485949000	1.305959000	-1.252149000
H	-0.497643000	2.163219000	0.302084000
H	-1.971204000	1.396011000	-0.303150000
H	-0.923649000	0.023251000	1.486680000
H	-0.577346000	-1.258007000	-1.264076000
H	-0.711484000	-2.122532000	0.274871000
H	-2.090378000	-1.202905000	-0.350275000
H	2.077872000	0.693067000	-0.474454000

Isomer 10

Conformer 10-1

ΔE=0.00

C	1.813337000	-0.622852000	-0.282452000
C	1.015783000	0.298776000	0.630062000
C	-0.130228000	0.976787000	-0.095291000
O	-1.033219000	0.054490000	-0.697988000
O	-1.652489000	-0.670449000	0.406884000
H	1.174115000	-1.405177000	-0.687414000
H	2.632111000	-1.096073000	0.257559000
H	2.238631000	-0.068571000	-1.120381000
H	0.615038000	-0.258081000	1.477086000

H	1.657269000	1.083393000	1.038761000
H	-0.682735000	1.647960000	0.567160000
H	0.233977000	1.543361000	-0.956043000
H	-2.576092000	-0.435404000	0.238184000

Conformer 10-2

ΔE=0.32

C	1.821197000	-0.616474000	-0.279189000
C	1.009054000	0.293677000	0.632645000
C	-0.131778000	0.975928000	-0.096971000
O	-1.047814000	0.066662000	-0.700392000
O	-1.770147000	-0.553716000	0.403866000
H	1.192483000	-1.391422000	-0.715599000
H	2.628925000	-1.102463000	0.266077000
H	2.262323000	-0.049612000	-1.099995000
H	0.603720000	-0.267087000	1.475329000
H	1.646315000	1.074936000	1.053565000
H	-0.683286000	1.650485000	0.561485000
H	0.237193000	1.542282000	-0.955465000
H	-1.534830000	-1.479475000	0.247899000

Conformer 10-3

ΔE=0.15

C	1.962974000	-0.699796000	0.134912000
C	1.280530000	0.611588000	-0.235079000
C	-0.141252000	0.700948000	0.283441000
O	-0.874082000	-0.316582000	-0.389656000
O	-2.205586000	-0.302064000	0.199745000
H	1.985580000	-0.828233000	1.217584000
H	2.989712000	-0.720703000	-0.227766000
H	1.431849000	-1.547704000	-0.292265000
H	1.831591000	1.456444000	0.182841000
H	1.269882000	0.744585000	-1.318388000
H	-0.180748000	0.518314000	1.360368000
H	-0.586171000	1.676603000	0.071314000
H	-2.717857000	-0.026579000	-0.574051000

Conformer 10-4

ΔE=0.39

C	2.314778000	-0.143505000	0.172809000
C	0.910363000	-0.337980000	-0.386934000
C	-0.050710000	0.666761000	0.214485000
O	-1.370344000	0.570465000	-0.308715000
O	-1.885755000	-0.713498000	0.154424000
H	2.689439000	0.857758000	-0.043642000
H	3.012039000	-0.859973000	-0.257997000
H	2.326207000	-0.277432000	1.255002000
H	0.908912000	-0.216100000	-1.470888000
H	0.551218000	-1.343586000	-0.170486000

H	-0.080723000	0.578129000	1.304620000
H	0.239522000	1.687246000	-0.047495000
H	-2.644415000	-0.393430000	0.663062000

Conformer 10-5

Δ*E*=0.17

C	-1.955918000	-0.709623000	0.133635000
C	-1.284546000	0.612466000	-0.218549000
C	0.141383000	0.702367000	0.287515000
O	0.871138000	-0.310183000	-0.396383000
O	2.249538000	-0.174476000	0.053265000
H	-1.432153000	-1.545902000	-0.325059000
H	-2.989202000	-0.726281000	-0.210031000
H	-1.958626000	-0.864010000	1.213417000
H	-1.283448000	0.764227000	-1.299000000
H	-1.837233000	1.445434000	0.220378000
H	0.585016000	1.675108000	0.066298000
H	0.189510000	0.525765000	1.366452000
H	2.355217000	-1.028331000	0.496882000

Conformer 10-6

Δ*E*=0.22

C	2.423170000	-0.303309000	0.013451000
C	1.156253000	0.544936000	-0.006788000
C	-0.076722000	-0.334359000	0.003026000
O	-1.196325000	0.541417000	-0.016689000
O	-2.369127000	-0.320467000	-0.089874000
H	2.462581000	-0.927279000	0.906421000
H	3.312107000	0.324927000	0.005238000
H	2.468315000	-0.958693000	-0.856358000
H	1.129777000	1.206498000	0.860018000
H	1.134849000	1.174486000	-0.896805000
H	-0.106405000	-0.980837000	-0.877624000
H	-0.108590000	-0.959680000	0.900213000
H	-2.785228000	-0.090627000	0.753273000

Conformer 10-7

Δ*E*=0.55

C	2.315845000	-0.139280000	0.156586000
C	0.900683000	-0.366944000	-0.362856000
C	-0.047525000	0.679042000	0.187480000
O	-1.376765000	0.574126000	-0.309674000
O	-1.942058000	-0.612904000	0.319804000
H	2.687821000	0.843067000	-0.136556000
H	3.004021000	-0.886670000	-0.234468000
H	2.344761000	-0.196299000	1.245031000
H	0.882902000	-0.318957000	-1.453116000
H	0.549572000	-1.355904000	-0.068351000
H	-0.070769000	0.653464000	1.280258000

H	0.251639000	1.680229000	-0.131369000
H	-2.113375000	-1.145617000	-0.469726000

Conformer 10-8

Δ*E*=1.42

C	1.556059000	-0.900896000	0.020536000
C	1.224081000	0.575484000	-0.158938000
C	-0.145778000	0.981896000	0.349860000
O	-1.228630000	0.431670000	-0.398501000
O	-1.407827000	-0.940106000	0.067366000
H	1.458228000	-1.197219000	1.065220000
H	2.579476000	-1.102307000	-0.294257000
H	0.887260000	-1.526433000	-0.565529000
H	1.956257000	1.186991000	0.376392000
H	1.292120000	0.851657000	-1.212830000
H	-0.276420000	0.727999000	1.404707000
H	-0.290460000	2.057355000	0.225540000
H	-2.320978000	-0.869452000	0.381089000

Conformer 10-9

Δ*E*=2.02

C	1.576101000	0.886359000	0.031316000
C	1.212356000	-0.578261000	-0.181379000
C	-0.155752000	-0.985564000	0.333965000
O	-1.253939000	-0.426819000	-0.382556000
O	-1.489351000	0.891245000	0.202530000
H	0.949689000	1.543680000	-0.567203000
H	2.613166000	1.066745000	-0.250156000
H	1.451729000	1.171819000	1.075754000
H	1.272126000	-0.832932000	-1.241429000
H	1.941048000	-1.210774000	0.333803000
H	-0.301052000	-2.059394000	0.200815000
H	-0.275180000	-0.748541000	1.393733000
H	-1.501431000	1.418785000	-0.608521000

Isomer 11

Conformer 11-1

Δ*E*=0.00

C	2.283254000	0.312245000	-0.007582000
C	0.990308000	-0.372483000	0.377159000
O	0.014942000	0.084326000	-0.552717000
O	-1.218491000	-0.600896000	-0.185537000
C	-2.098128000	0.433311000	0.219250000
H	2.567362000	0.048186000	-1.024149000
H	3.077298000	-0.002631000	0.667996000
H	2.180730000	1.393657000	0.057773000
H	1.074008000	-1.458653000	0.307293000
H	0.681970000	-0.107455000	1.392073000

H	-1.707971000	0.963133000	1.089871000
H	-2.273108000	1.134649000	-0.596250000
H	-3.024500000	-0.076767000	0.478464000

Conformer 11-2

Δ*E*=0.15

C	1.648148000	0.888026000	-0.025261000
C	1.257288000	-0.571315000	0.074222000
O	-0.057737000	-0.827345000	-0.406695000
O	-0.944158000	-0.222595000	0.586721000
C	-1.903252000	0.482601000	-0.179240000
H	1.582758000	1.230851000	-1.056636000
H	2.673028000	1.023722000	0.320746000
H	0.995021000	1.498514000	0.594462000
H	1.876880000	-1.196733000	-0.570110000
H	1.338557000	-0.939530000	1.099245000
H	-1.437202000	1.290891000	-0.744149000
H	-2.428495000	-0.190403000	-0.857109000
H	-2.598493000	0.886340000	0.555015000

Conformer 11-3

Δ*E*=0.23

C	2.004813000	-0.535041000	0.026138000
C	1.047913000	0.598541000	0.325800000
O	-0.078494000	0.612328000	-0.546020000
O	-0.849739000	-0.578073000	-0.200832000
C	-2.097768000	-0.072454000	0.240175000
H	1.515697000	-1.494124000	0.178073000
H	2.869330000	-0.477020000	0.687881000
H	2.349013000	-0.475643000	-1.004707000
H	0.701120000	0.567290000	1.362253000
H	1.509906000	1.569833000	0.140528000
H	-1.978462000	0.547598000	1.130015000
H	-2.584499000	0.499177000	-0.549653000
H	-2.685988000	-0.957425000	0.477748000
